# *Helicobacter pylori* Cholesteryl α-Glucosides Contribute to Its Pathogenicity and Immune Response by Natural Killer T Cells

**DOI:** 10.1371/journal.pone.0078191

**Published:** 2013-12-02

**Authors:** Yuki Ito, Jose Luis Vela, Fumiko Matsumura, Hitomi Hoshino, Aaron Tyznik, Heeseob Lee, Enrico Girardi, Dirk M. Zajonc, Robert Liddington, Motohiro Kobayashi, Xingfeng Bao, Jeanna Bugaytsova, Thomas Borén, Rongsheng Jin, Yinong Zong, Peter H. Seeberger, Jun Nakayama, Mitchell Kronenberg, Minoru Fukuda

**Affiliations:** 1 Cancer Center, Sanford-Burnham Medical Research Institute, La Jolla, California, United States of America; 2 Del E. Webb Neuroscience, Aging and Stem Cell Research Center, Sanford-Burnham Medical Research Institute, La Jolla, California, United States of America; 3 Infectious and Inflammatory Disease Center, Sanford-Burnham Medical Research Institute, La Jolla, California, United States of America; 4 La Jolla Institute for Allergy & Immunology, La Jolla, California, United States of America; 5 Department of Molecular Pathology, Shinshu University Graduate School of Medicine, Matsumoto, Nagano, Japan; 6 Department of Medical Biochemistry and Biophysics, Umeå University, Umeå, Sweden; Faculdade de Medicina, Universidade de São Paulo, Brazil

## Abstract

Approximately 10–15% of individuals infected with *Helicobacter pylori* will develop ulcer disease (gastric or duodenal ulcer), while most people infected with *H. pylori* will be asymptomatic. The majority of infected individuals remain asymptomatic partly due to the inhibition of synthesis of cholesteryl α-glucosides in *H. pylori* cell wall by α1,4-GlcNAc-capped mucin *O*-glycans, which are expressed in the deeper portion of gastric mucosa. However, it has not been determined how cholesteryl α-glucosyltransferase (αCgT), which forms cholesteryl α-glucosides, functions in the pathogenesis of *H. pylori* infection. Here, we show that the activity of αCgT from *H. pylori* clinical isolates is highly correlated with the degree of gastric atrophy. We investigated the role of cholesteryl α-glucosides in various aspects of the immune response. Phagocytosis and activation of dendritic cells were observed at similar degrees in the presence of wild-type *H. pylori* or variants harboring mutant forms of αCgT showing a range of enzymatic activity. However, cholesteryl α-glucosides were recognized by invariant natural killer T (*i*NKT) cells, eliciting an immune response *in vitro* and *in vivo*. Following inoculation of *H. pylori* harboring highly active αCgT into *i*NKT cell-deficient (Jα18^−/−^) or wild-type mice, bacterial recovery significantly increased in Jα18^−/−^ compared to wild-type mice. Moreover, cytokine production characteristic of Th1 and Th2 cells dramatically decreased in Jα18^−/−^ compared to wild-type mice. These findings demonstrate that cholesteryl α-glucosides play critical roles in *H. pylori*-mediated gastric inflammation and precancerous atrophic gastritis.

## Introduction

The gastric pathogen *Helicobacter pylori* is a bacterium that infects over 50 percent of the world's population [1]. If untreated, this infection leads to chronic gastritis and development of pyloric gland atrophy, peptic ulcer, intestinal metaplasia, gastric carcinoma, and mucosa-associated lymphoid tissue (MALT) lymphoma [2].

The initial host response to *H. pylori* is strong neutrophilic recruitment, which leads to gastric epithelial damage and is followed by chronic inflammation [3], [4]. Such chronic inflammation is associated with infiltration of lymphocytes and plasma cells, forming MALT. In this process, venules in the gastric lamina propria begin to exhibit a high-endothelial venule (HEV)-like phenotype, which likely facilitates immune cell infiltration. Indeed, we have shown that induction of HEV-like vessels is associated with recruitment of mononuclear cells to inflammatory sites, and that eradication of *H. pylori* with antibiotics and treatment with proton pump inhibitors leads to disappearance of HEV-like vessels and diminished mononuclear cell infiltration [3].

After infection, *H. pylori* primarily colonizes surface mucosa of the stomach and rarely reaches deeper portions of the gastric mucosa [5], [6], although a more invasive and intracellular infection has also been proposed [7]. Gastric mucins are divided into surface and gland mucins [8]. The latter, consists of MUC6, are found in deeper regions of the stomach and are characterized by expression of α1,4-linked *N*-acetylglucosamine (α1,4-GlcNAc) attached to core 2-branched *O*-glycans, which is absent in the surface mucin, MUC5AC [6], [9]. It is known that MUC6 is exclusively expressed in mucous neck cells and pyloric glands of the gastric mucosa, while MUC5AC is expressed in gastric surface mucous cells in the stomach [10]. These two types of mucins form a surface mucous gel layer exhibiting an alternating laminated array [11]. Since this differential distribution coincides with distribution of *H. pylori*, we previously examined the antibiotic activity of α1,4-GlcNAc mucin and found that α1,4-GlcNAc-containing mucins inhibit *H. pylori* growth by blocking synthesis of cholesteryl α-glucosides [12], the major component of *H. pylori* cell wall lipids [13]. Moreover, mutant mice deficient in α1,4-*N*-acetylglucosaminyltransferase exhibit adenocarcinoma, indicating that α1,4-GlcNAc-containing mucins function as tumor suppressors [10]. Significantly, *H. pylori* lacks cholesterol and must incorporate it from surrounding host epithelial cells [14]. Cholesteryl α-glucosyltransferase (αCgT) adds an α-glucosyl residue to cholesterol [15], forming cholesteryl α-glucoside (αCGL). αCGL is further derivatized in *H. pylori* to form cholesteryl acyl α-glucoside (αCAG), cholesteryl phosphatidyl α-glucoside (αCPG), and cholesteryl phosphatidyl monoacyl α-glucoside (αCPG (monoacyl)) [13]. We previously cloned αCgT using the shotgun method [16] and showed that its activity is inhibited by core 2 *O*-glycan capped by α1,4-GlcNAc residues [17]. However, the function of cholesteryl α-glucosides in the pathogenesis of *H. pylori* infection has not been determined.

Invariant natural killer T (*i*NKT) cells are recognized as immune cells that react with glycolipids. *i*NKT cells express the T cell receptor (TCR) encoded by Vα24-Jα18 and Vα14-Jα18 rearrangements in human and mouse, respectively [18], [19]. These TCRs recognize glycolipid antigen presented by CDld, a non-classical MHC class I-like antigen distinct from c-type lectins that mediate leukocyte and lymphocyte adhesion [20], [21]. *i*NKT cells exhibit unique aspects of both innate and adaptive immunity, distinguishing them from innate immune natural killer (NK) cells [22]–[24]. Both activated *i*NKT and NK cells can rapidly produce large amounts of various cytokines such as interleukin (IL)-4, interferon-γ (IFN-γ), tumor necrosis factor-α (TNF-α) and IL-17, which likely stimulate different immune cell populations with diverse functions [19]. The potent TCR antigen of *i*NKT cells is α-galactosylceramide but also includes galactosyl diacyl glycerol present in Lyme disease-causing *Borrelia burgdorferi* [25], [26], α-galacturonic ceramide from *Sphingomonas spp* [27], and α-glucosyldiacylglycerol from *Streptoccocus pneumonia* [28]. However, how *i*NKT cells respond to these bacteria during the course of infection in human patients is not known.

Here, to characterize αCGL function in the innate immune response, we first isolated the αCgT gene from *H. pylori* retrieved from stomach tissues of *H. pylori*-infected patients. We found that the activity of cloned αCgT from clinical isolates positively correlates with the atrophy score of stomach tissue. We then constructed recombinant *H. pylori* harboring αCgT from different clinical isolates and found that αCgT activity is positively correlated with susceptibility to *i*NKT cells. Moreover, *H. pylori* containing highly active αCgT were recovered from *i*NKT cell-deficient mice at levels dramatically higher than from wild-type (WT) mice. *In vitro* and *in vivo* analysis identified αCPG (monoacyl) is the most potent antigen for *i*NKT cells among *H. pylori* cell components. These findings demonstrate that cholesteryl α-glucosides induce an immune response by *i*NKT cells, thus causing stomach inflammation due to *H. pylori* infection.

## Results

### *H. pylori* αCgTs isolated from Japanese patients show varying levels of activity relative to αCgT from *H. pylori* 26695

**Figure 1:**
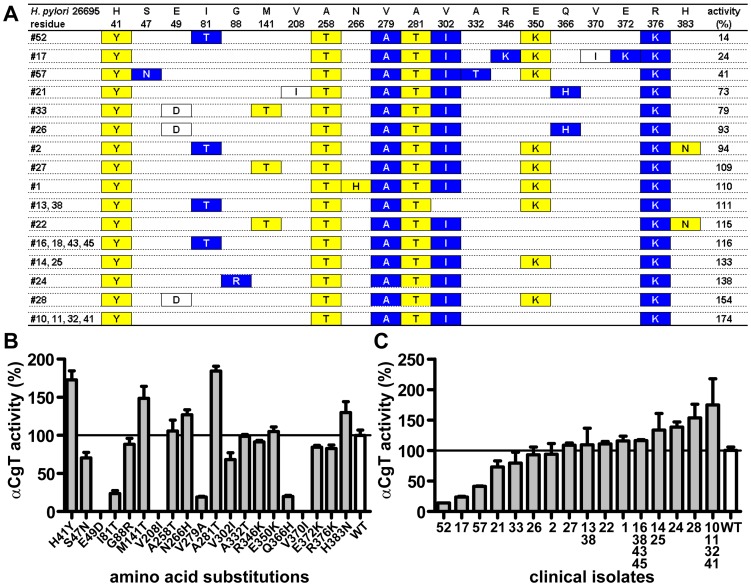
Amino acid sequences of αCgT from clinical isolates and αCgT activity of protein variants. (A) The αCgT amino acid sequence from 24 clinical isolates was compared to that of *H. pylori* 26695, whose whole genome sequence has been reported. Only variant residues are shown. Residues in yellow and blue represent substitutions that yield higher and lower αCgT activity, respectively, relative to αCgT from *H. pylori* 26695. Proteins, of which amino acid residues shown in white boxes were substituted, were not soluble as a recombinant protein in a bacterial expression system and therefore enzyme activity was not assayed. (B) cDNA encoding the amino acid sequence of αCgT from *H. pylori* 26695 in an expression vector was mutated by site-directed mutagenesis to reproduce residues seen in *H. pylori* clinical isolates. Bacterially expressed αCgT was assayed using [^3^H]UDP-glucose and cholesterol as described in File S1. (C) The entire αCgT sequence in the expression vector was replaced with sequences from *H. pylori* clinical isolates and activity of expressed αCgT was assayed. The assay was performed in triplicate and repeated twice in both (A) and (B). Representative results are shown.

To determine the role of cholesteryl α-glucosides in *H. pylori* pathogenesis in the stomach, αCgT genomic DNA was isolated from clinical *H. pylori* isolates from the stomachs of 24 *H. pylori*-infected Japanese patients. Amino acid sequences deduced from various αCgT genomic sequences showed at least 20 different amino acid substitutions compared to αCgT from control WT *H. pylori* 26695, whose whole genome has been sequenced [29] (Figure 1A). DNA encoding αCgT *H. pylori* 26695 WT was mutated by site-directed mutagenesis to create sequences corresponding to clinical isolates, and mutant proteins were expressed in a bacterial expression vector [30] and their activities measured. Some enzymes showed activity higher than WT αCgT from *H. pylori* 26695, while others showed decreased activity (Figure 1B), as indicated in yellow and blue, respectively, in Figure 1A.

The amino acid sequence of αCgTs derived from clinical isolates of 18 European and 5 Indian patients was also determined (data not shown). A tyrosine substitution for WT histidine at position 41, which is an activating mutation, is observed in all Japanese isolates; that mutation was only occasionally seen in isolates of European and Indian origin (data not shown), indicating that protein sequences from Japanese patients are more uniform than those isolated from Indian and European individuals. Moreover, all *H. pylori* isolates from Japanese patients harbored genes encoding the most toxic form of *cagA* and *vacA* (*cagA*-positive and *vacA* s1/m1, data not shown) [31]–[33]. However, more than half of the Indian and European clinical isolates harbored the much less toxic *vacA* s1/m1 or non-toxic *vacA* s2/m2, and about a quarter of the European *H. pylori* specimens lacked *cagA* (data not shown). Due to this diversity, for the remainder of the experiments reported here, we analyzed *H. pylori* from Japanese patients only.

To determine the effect of amino acid substitutions seen in different *H. pylori* clones, the entire αCgT sequence in *H. pylori* 26695 was replaced with sequences present in the 16 different patterns of substitutions of αCgT amino acids and expressed in *Escherichia coli*, and the mutant αCgT proteins were purified. The activity of those recombinant proteins showed significant variation among clinical isolates, and more than half of the αCgT variants showed increased activity relative to WT *H. pylori* (Figure 1C).

**Figure 2:**
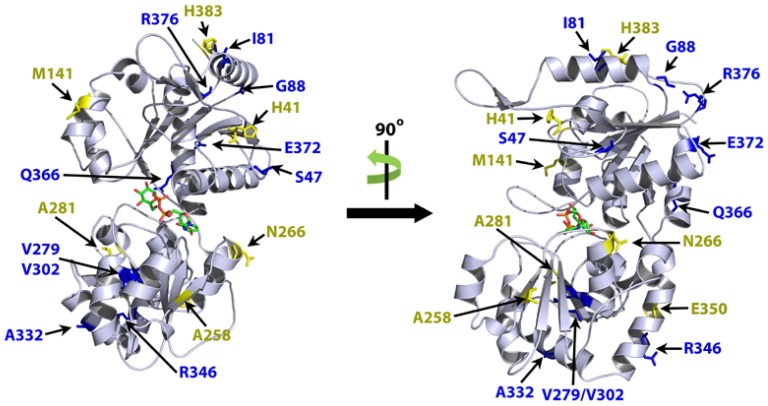
Ribbon model of αCgT adapted from crystal structure of phosphatidylinositol mannosyltransferase (PimA) from *Mycobacterium smegmatis*. Homology modeling of αCgT was performed by alignment of αCgT in *H. pylori* 26695 (wild type) and PimA in *M. smegmatis*. Amino acid substitutions observed in Japanese clinical isolates that increase (yellow) and decrease (blue) enzyme activity relative to WT αCgT are shown. The figures are adapted from a figure previously reported in [35].

To determine potential effects of amino acid substitutions on αCgT structure, we firstly attempted to determine the αCgT crystal structure. However, since we could not accomplish this task due to αCgT hydrophobicity, we searched databases for enzymes of similar structure [34]. Our search clearly identified αCgT as a member of the GT-4 family. GT-4 proteins exhibit two Rossmann-fold domains with the active site in a cleft between the two domains. The best hit with a known 3-dimensional structure was phosphatidylinositol mannosyltransferase from *Mycobacterium smegmatis* (PDB code 2GEJ) [35]. In this case, sequence identity was only 17%, but a “Z-score” of −65.2 indicated high structural similarity (a value of −9.5 indicates 97% confidence) [34]. There was only one major gap in the primary sequence of αCgT: a 12-residue insertion in a loop far from the GDP-mannose binding site. We next built a 3-dimensional model using 2GEJ as template (Figure 2). The model was of high quality as judged by the distribution of hydrophobic residues in the protein core and hydrophilic residues on the surface. Moreover, analysis of the UDP-glucose binding pocket revealed that most of the critical binding residues were identical, including residues implicated in catalysis [35]. Notably, all of the αCgT amino acid substitutions from Japanese patients are located in the surface region of the αCgT sterical structure and are absent in the UDP-Glc binding pocket (Figure 2).

**Figure 3:**
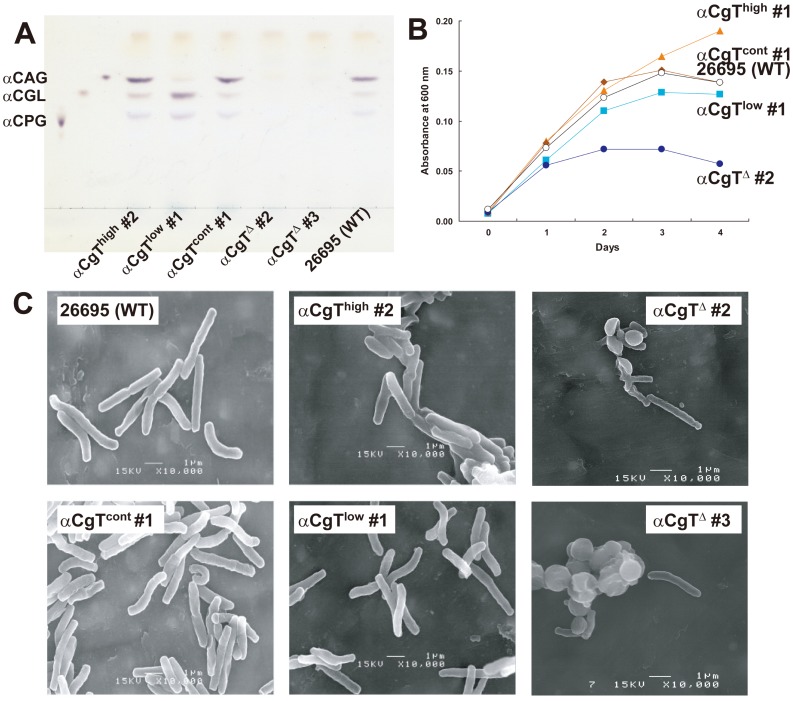
Characterization of *H. pylori* expressing αCgT enzymes of varying activity. (A) Thin-layer chromatography (TLC) analysis of αCGL, αGAG, and αGPG from *H. pylori* expressing αCgT showing high activity (αCgT^high^), low activity (αCgT^low^), control activity (αCgT^cont^), no activity (αCgT^Δ^) and WT activity (*H. pylori* 26695 (WT)). Cholesteryl α-glycosides were detected by orcinol-sulfuric acid reagent after TLC. (B) Growth curve of various *H. pylori* clones as measured by absorbance at 600 nm. The experiment was done in duplicate and a representative result is shown. Note that a *H. pylori* expressing the lowest αCgT activity recombinant protein and a clone derived from Japanese clinical isolate #52 grew similarly to αCgT^low^. (C) Scanning electron photomicrographs of the different *H. pylori* clones described above. Photomicrographs were taken at 10,000-fold magnification. Scale bar = 1 µm.

We next used homologous recombination to replace the *H. pylori* 26695 αCgT sequence with sequences from *H. pylori* harboring αCgT of higher (αCgT^high^, strain #10) and lower (αCgT^low^, strain #17) activity or to create *H. pylori* lacking the αCgT gene altogether (αCgT^Δ^) in order to compare these variants with parental *H. pylori* 26695 (αCgT^cont^) (Figure S1 and Table S1 in File S1). As anticipated, *H. pylori* αCgT^high^ synthesized greater amounts of cholesteryl α-glucosides than did *H. pylori* αCgT^low^, while *H. pylori* lacking αCgT synthesized no cholesteryl α-glucosides (Figure 3A). Among different cholesteryl α-glucosides, αCGL was the most abundant in products of *H. pylori* αCgT^low^, most likely because αCGL was not converted to αCAG or αCPG.

Significantly, the αCgT^Δ^ *H. pylori* strain grew much more slowly and entered plateau phase earlier than did the parental *H. pylori* 26695 (WT *H. pylori*), and the αCgT^high^ *H. pylori* clone grew faster than the WT form in liquid culture (Figure 3B). Electron microscopic analysis showed that some αCgT^Δ^ *H. pylori* exhibited an aberrant coccoid form [36], [37] (Figure 3C). These results indicate that cholesteryl α-glucosides are critical for *H. pylori* growth and normal morphology.

### *H. pylori* αCgT activity is highly correlated with stomach atrophy

**Figure 4:**
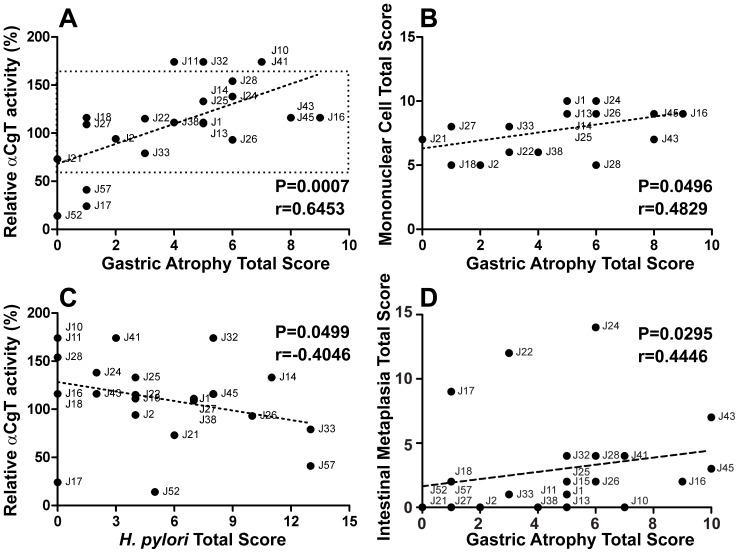
Relationship of αCgT activity to total gastric atrophy and recruitment of mononuclear cells. (A, C) Relationship of αCgT activity to total gastric atrophy score based on 24 stomach tissue samples from which *H. pylori* was isolated (A), and correlation of enzyme activity with total number of *H. pylori* isolated from stomach tissue. (B) Correlation of the total number of mononuclear cells with gastric atrophy based on analysis of 17 samples shown within the dotted square in Fig. 4A. Patient numbers are the same as in Figure 1. Statistical significance was assessed using Prism 5 and evaluated by Spearman's rank correlation coefficient method. (C). (D) Shown is the relationship between intestinal metaplasia and gastric atrophy.

We then asked if αCgT levels, and thus those of cholesteryl α-glucosides, were correlated with pathogenesis of *H. pylori* infection. Histological grading of 24 human biopsy samples of the gastric mucosa collated together with the 24 Japanese *H. pylori* strains were judged by five different criteria using the updated Sydney System [38]: *H. pylori* infection load, recruitment of neutrophils, infiltration of mononuclear cells, glandular atrophy (antrum and corpus), and intestinal metaplasia, as illustrated in Figure S2A and B. These criteria were evaluated each as four grades scored from 0 to 3: normal (score 0), mild (1), moderate (2), and marked (3). In general, more advanced atrophy was characterized by a decrease in the number of pyloric glands in the antrum and fundic glands in the corpus (data not shown). More advanced atrophy was also associated with recruitment of mononuclear cells and formation of intestinal metaplasia (data not shown). Histological assessment of the gastric mucosa was undertaken based on pathology reports, which were reviewed by one senior pathologist (J.N., Figure S2B). When αCgT activity of all strains was plotted against these parameters, activity was highly correlated with the total atrophy score (antrum and corpus) (Figure 4A). Replotting of those data, as indicated by the dotted rectangle in Figure 4A, showed that the total atrophy score is positively correlated with mononuclear cell recruitment (Figure 4B). Moreover, the number of *H. pylori* colonies was inversely correlated with αCgT activity (Figure 4C). These results strongly suggest that cholesteryl α-glucosides induce an immune response causing increased inflammation, yet that response decreases the number of surviving *H. pylori*. Furthermore, the atrophy score was correlated with intestinal metaplasia (Figure 4D), a precancerous phenotype [39], indicating that a high atrophy score predicts progression to gastric carcinoma.

### Cholesteryl α-glucosides are responsible for an *i*NKT cell immune response

To determine which immune cells play a critical role in *H. pylori* infection, we first assayed phagocytosis by macrophage-like differentiated THP-1 cells (Figure S3A) and antigen presentation by dendritic cells (DCs) (Figure S3B). Phagocytosis was observed in all αCgT clones, regardless of αCgT activity (Figure S3A). Activation of DCs was measured by the expression of 3 markers, CD86, HLA-DR, and CD40. CD86 and HLA-DR are known antigen-presentation markers and CD40 is a differentiation marker of mature DCs. None of αCgT clones exhibited a significant difference in terms of recognizing *H. pylori* expressing αCgT^high^, αCgT^low^, or αCgT^Δ^, in antigen presentation by DCs (Figure S3B). This result suggests that generally all αCgT clones analyzed are similarly recognized by macrophages and DCs.

**Figure 5:**
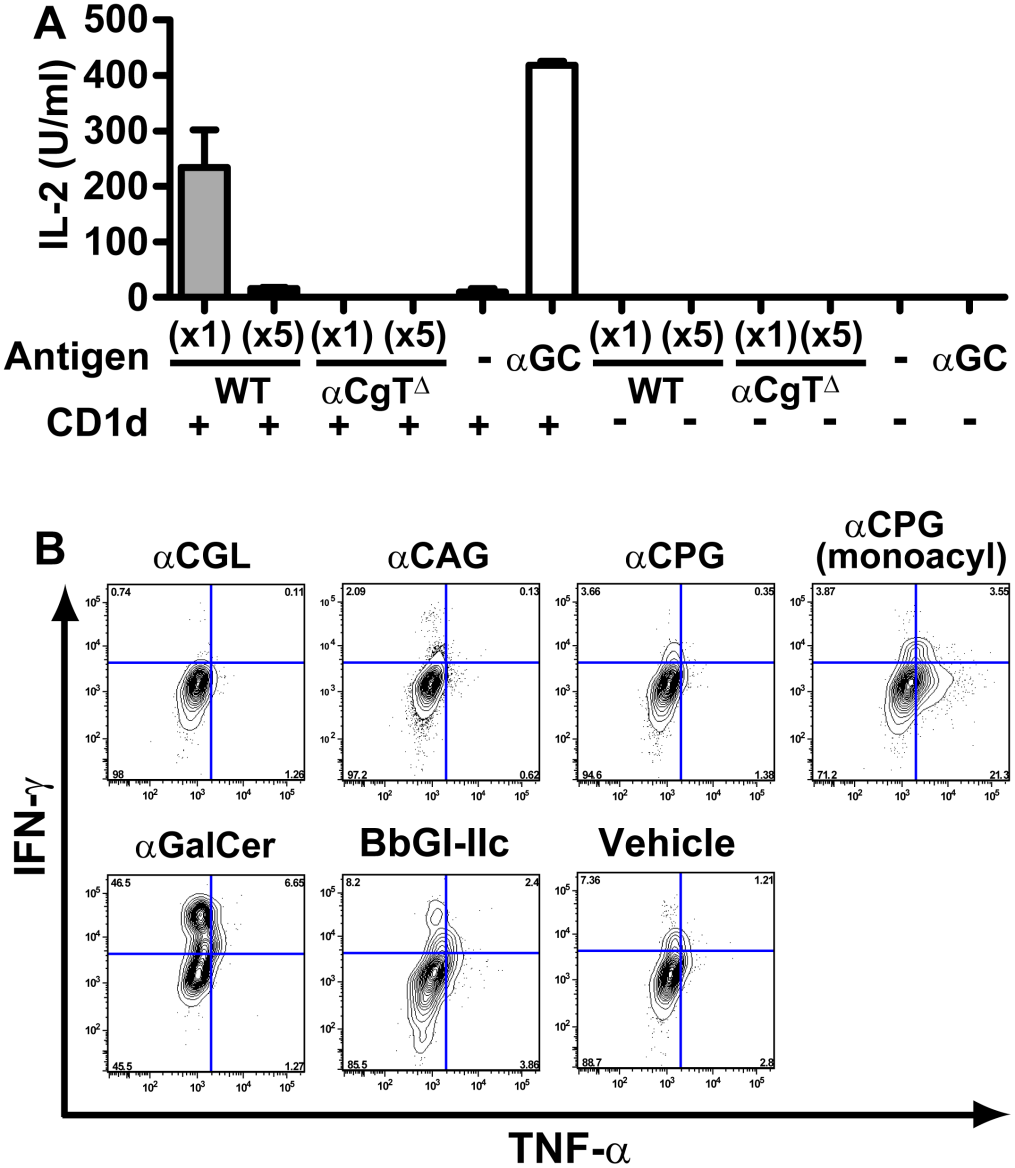
*In vitro* and *in vivo* responses to *i*NKT cell activation. (A) *H. pylori* lysates from wild type (WT) or αCgT^Δ^ clones at 10^7^ CFU/well (×1) or 5-fold diluted (×5) were incubated with 10 µg/ml mouse CD1d-tetramer for 24 hours and then with mouse *i*NKT cells for another 16 hours prior to analysis of IL-2 in supernatants. α-galactosylceramide as antigen (6 ng/well) served as a positive control. WT *H. pylori* and α-galactosylceramide produced equivalent levels of IL-2. IL-2 production was absent when CD1d was absent. Means ± S. D. are shown. (B) Mouse DCs were isolated from bone marrow, activated with the indicated cholesteryl α-glycosides (50 µg/ml) or vehicle, and injected into WT mice. Sixteen hours later, liver mononuclear cells were subjected to FACS analysis to assay IFN-γ and TNF-α in α-galactosylceramide/CD1d-gated cells. α-galactosylceramide (αGalCer) and BbGL-IIc glycolipid from *B. burgdorferi* served as positive antigens.

We then tested the possibility that *i*NKT cells exert a differential response to *H. pylori* expressing αCgT^high^ or αCgT^Δ^. Upon recognition of WT *H. pylori*, a mouse hybridoma *i*NKT cell line produced significant amounts of IL-2, an indicator of *i*NKT cell activation. Those levels were roughly equivalent to stimulation by α-galactosylceramide, a bona fide antigen for *i*NKT cells. By contrast, *H. pylori* lacking αCgT were barely recognized by the same *i*NKT hybridoma based on failure to elicit an IL-2 response (Figure 5A). These results indicate that *i*NKT cell immune responses are largely due to recognition of cholesteryl α-glucosides.

Cholesteryl α-glucosides constitute 25% of total *H. pylori* lipids and comprise three major forms and one minor form (Figure S4) [13]. All four forms were synthesized and their structure confirmed by NMR. When we evaluated the three major forms of synthetic cholesteryl α-glucosides *in vitro*, cholesteryl phosphatidyl α-glucoside (αCPG) elicited the highest *i*NKT cell response when glycolipids were initially dissolved in DMSO (Figure S5A), although the response toward αCPG was significantly less robust than to α-galactosylceramide. This observation is consistent with a generally weak interaction of the glycolipid with CD1d (Figure S5B) and/or the T-cell receptor. Cholesteryl β-glucoside (βCGL) was not recognized by *i*NKT cells (Figure S5A). In other experiments, DCs were isolated from bone marrow and differentiated using GM-CSF. These immature DCs were then incubated with the four synthetic forms of cholesteryl α-glucosides and injected intraperitoneally into WT mice. The presence of *i*NKT cells in liver, where they are more abundant than in other tissues, was evaluated 16 hours later. Interestingly, the monoacylated form of αCPG, which is reportedly a minor component of cholesteryl α-glucosides [40], was the most potent antigen in the *in vivo* assay (Figure 5B). Consistently, an isoelectrofocusing assay showed that αCPG (monoacyl) was the only lipid that interacted with CD1d (Figure S5B). These results suggest that fatty side chain(s) in cholesteryl α-glucosides are potentially important for CD1d recognition.

### *H. pylori* growth increases in Vα14 *i*NKT cell-deficient mice

**Figure 6:**
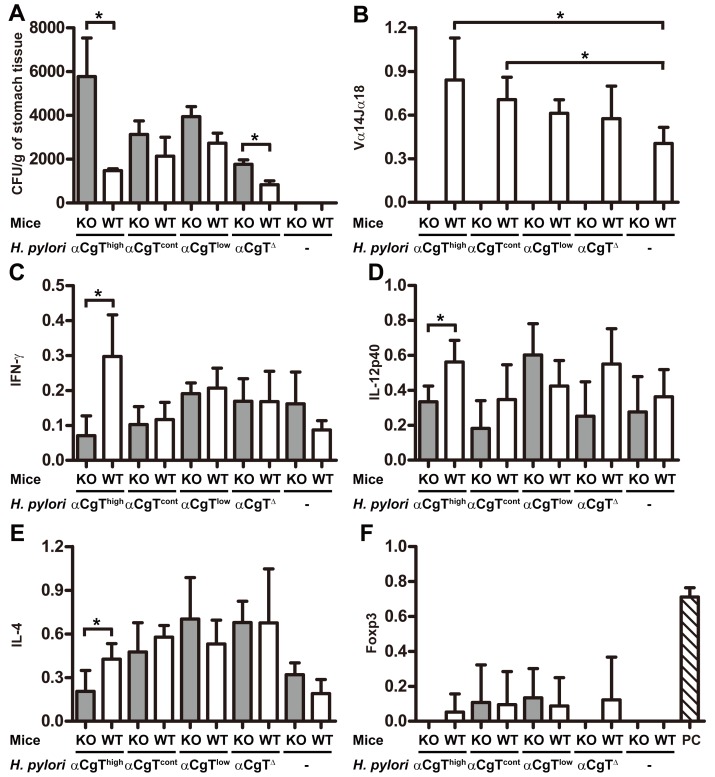
*H. pylori* survival in WT and Jα18^−/−^ mice and gene expression analysis of chemokines and transcription markers. The stomachs of WT (n = 4) or Jα18^−/−^ (KO) (n = 4) mice infected with *H. pylori* expressing αCgT variants were assayed for bacterial number and host response 10 days later. (A) Bacterial recovery from the stomach of *H. pylori*-infected WT or Jα18^−/−^ mice. (B) Expression of Vα14-Jα18 transcripts in stomach tissue specimens from WT mice as measured by RT-PCR. Tissue specimens are the same as those shown in panel A. (C, D, E, F) Expression of IFN-γ (C), IL-12p40 (D), IL-4 (E), and Foxp3 (F) as assayed by RT-PCR. The same cDNA samples used in panel B were used in C through F. Transcript levels were normalized to the β-actin signal. PC in (F) stands for positive control for Foxp3. Statistical analysis was performed using Prism 5 and an unpaired *t*-test (*, *P*<0.05). Means ± S. D. are shown.

To determine the role of *i*NKT cells in the *in vivo* immune response to cholesteryl α-glucosides, *H. pylori* clones expressing different αCgTs were inoculated into the stomach of WT or Vα14 *i*NKT-cell knockout (Jα18^−/−^) mice, which were generated by genetic deletion of a T cell receptor (Vα14) that recognizes CD1d-bound glycolipids and is unique to *i*NKT cells [41]. Ten days after the 3rd inoculation, mice were sacrificed and the stomach was excised. Previous reports indicate that macrophage and neutrophil recruitment subsides by 10 days after *H. pylori* inoculation, while T lymphocyte recruitment is initiated 10 days after inoculation [42]. Indeed, histochemical analysis showed that surface mucosa from *H. pylori*-infected Jα18^−/−^ and WT mice was indistinguishable and only a few mononuclear cells, neutrophils or macrophages had been recruited by the 10 day time point (Figure S6A and B). Under these same conditions, αCgT^high^ *H. pylori* were recovered at lower levels from the stomach of WT mice than from αCgT^cont^ *H. pylori*-infected WT mice. Furthermore, recovery of αCgT^high^ *H. pylori* was five times greater in Jα18^−/−^ than in WT mice (Figure 6A). Such a substantial increase relative to WT mice was not observed when αCgT^Δ^ *H. pylori* were inoculated into Jα18^−/−^ mice. Significantly, increased mRNA expression of *i*NKT cells (Table S2 in File S1) were expressed in the stomach and/or underwent proliferation upon inoculation of αCgT^high^ *H. pylori* and WT *H. pylori* relative to controls (Figure 6B). Significantly, a greater number of transcripts expressed in *i*NKT cells were present in stomach tissue derived from patients infected with *H. pylori* than in control samples, based on quantitative real-time PCR analysis of polyA^+^ RNA isolated from those tissue specimens (Figure S7). Overall, these results suggest that *H. pylori* containing cholesteryl α-glucosides induce proliferation and/or recruitment of *i*NKT cells to the stomach, where they attack *H. pylori* in infected tissue.

To evaluate the consequences of an *i*NKT response upon *H. pylori* infection, we examined levels of mRNAs encoding cytokines. Following infection of mice with αCgT^high^ bacteria, IFN-γ, IL-12p40, and IL-4 transcript levels were significantly decreased in Jα18^−/−^ relative to WT mice (Figure 6C, D, and E). Similarly, expression of IL-2, IL-5, IL-10, and lymphotoxin (LT)-β was reduced in Jα18 knockout mice (Figure S8A, B, D, and F). These results indicate that *i*NKT cell activation by *H. pylori* cholesteryl α-glucosides promotes proliferation and/or recruitment of cells producing Th1 cytokines (IFN-γ, IL-2, and IL-12p40) [43], Th2 cytokines (IL-4, IL-5), and a regulatory cytokine (IL-10) [44]. Th17 cells, which are marked by RORγt expression [45], are reportedly associated with an autoimmune responses [46]. We also observed increased RORγt expression as well as that of IL-10, which encodes an inducer of regulatory T cells (Tregs), in stomach tissue from WT infected mice, both indicators of *i*NKT cell activation (Figure S8E and G). However, Foxp3 [47] (Table S2 in File S1) and IL-22 transcripts were hardly detectable, even following analysis using different sets of RT-PCR primers (Figure 6F and Figure S8H), indicating that Tregs were not activated. These results indicate that recognition of cholesteryl α-glucosides by *i*NKT cells stimulates immune cell responses in various cell lineages, including Th1, Th2, and Th17 cells, and that these responses are associated with decreased recovery of *H. pylori*.

## Discussion

Infection of the stomach with *H. pylori* induces an acute immune response mediated predominantly by neutrophil infiltration, but the subsequent innate response promotes chronic inflammation mediated by various immune cells. This long-term phase apparently induces HEV-like vessels in gastric mucosa, facilitating T and B lymphocyte recruitment to inflammatory sites in the stomach. We previously found that the presence of *H. pylori* in the stomach is necessary to facilitate lymphocyte recruitment, and that *H. pylori* eradication abrogated HEV-like vessels and dramatically decreased the number of mononuclear cells [3]. Similarly, we found recently that gastric MALT lymphoma is associated with the appearance of HEV-like vessels that express MECA-79 negative sialyl Lewis X [48], suggesting that non-sulfated sialyl Lewis X recruits lymphocytes during progression of gastric MALT-lymphoma.

Here we show that cholesteryl α-glucosides in *H. pylori* play a critical role in the early phase of inflammation in *H. pylori*-infected mice. Moreover, we observed highly significant diversity in αCgT amino acid sequences depending on clinical isolates and found that αCgT activity was highly correlated with progression of gastric mucosal atrophy. αCgT amino acid substitutions were not seen at the UDP-Glc binding site, which is located at an inner hydrophobic pocket of αCgT. This observation supports our conclusion that αCgT is essential for *H. pylori* growth and that mutations that alter UDP-Glc binding abolish *H. pylori* viability. Amino acid substitutions that we observed in αCgT from the clinical isolates likely result in αCgT conformational changes, resulting in increased or decreased αCgT activity.

It has been reported that *H. pylori* is one of the most diverse and variable bacterial species studied. Genetic variation can be generated in a bacterial population by mutation and/or recombination between different strains. As a result, *H. pylori* exhibits extensive genetic variation, so that almost every infected individual carries their own *H. pylori* clinical strain. [49]. To investigate the effect of genomic diversity on αCgT activity, we analyzed 24 clinical isolates from Japanese patients, as well as samples from 5 Indian and 18 Europe individuals. Genotyping analysis of *H. pylori cagA* and *vacA*, which encode toxic factors [31]–[33], showed that isolates from all Japanese patients contained toxic forms of these genes (*cagA*-positive, *vacA*; s1/m1), while some *H. pylori* isolates from European and Indian patients showed *cagA*-negative or weakly toxic or non-toxic *vacA* subtypes. Moreover, substitution of the histidine residue at position 41 with tyrosine activated αCgT relative to wild-type *H. pylori*. That substitution was observed in all clinical isolates from Japanese patients, while only a fraction of European and Indian patients harbored that substitution. This finding, together with the presence of CagA and the toxin known as VacA, may account for the high prevalence of gastric cancer in Japan. Additional large-scale examination of *H. pylori* isolates from patients in different countries such as European nations is necessary to support this hypothesis.

The present study revealed that αCgT activity is higher in *H. pylori* isolated from patient clinical isolates. As biospecimens were isolated upon diagnosis, the specimens used in this study came from patients who had been infected for varying periods of time. It is noteworthy that αCgT activity is highly correlated with glandular atrophy, regardless of infection history. The degree of atrophy is also correlated with intestinal metaplasia, a putative precancerous condition, supporting the idea that the inflammatory response leads to gastric cancer [50]. Moreover, unlike normal gastric mucosa, which shows stable expression of α1,4-GlcNAc residues in gastric glands, it is reported that expression of α1,4-GlcNAc residues containing *O*-glycans in *H. pylori*-associated intestinal metaplasia is significantly reduced [51]. This fact suggests that disappearance of core 2 *O*-glycans capped by α1,4-GlcNAc residues may function in the process of intestinal metaplasia. Although previous studies demonstrate a role for *i*NKT cells in various chronic infection-inflammation states, our work demonstrates that *i*NKT cell-mediated chronic inflammation is directly correlated to disease progression in human patients.

Here, we generated recombinant *H. pylori* in which only the αCgT gene was replaced with forms seen in clinical isolates. We found that *H. pylori* harboring αCgT^high^ grows more efficiently than does *H. pylori* expressing αCgT^low^. Moreover, some *H. pylori* lacking αCgT exhibited a coccoid morphology. These results demonstrate that αCgT is critical for *H. pylori* growth. However, forms of *H. pylori* that express different amounts of cholesteryl α-glucosides did not induce a differential response toward macrophages and DCs, supporting the idea that cholesteryl α-glucosides are antigens recognized by *i*NKT cells. Notably five-fold greater levels of *H. pylori* αCgT^high^ were recovered from Jα18^−/−^ than from WT mice. This difference was much greater than differences observed in WT and Jα18^−/−^ mice infected with either αCgT^cont^ or αCgT^Δ^ *H. pylori*. These results clearly show that excess cholesteryl α-glucosides are recognized by *i*NKT, reducing *H. pylori* infection. Our findings differ from a previous report showing that cholesteryl α-glucosides protect *H. pylori* from immune cell attack [14]. That work relied on only one *H. pylori* mutant lacking cholesteryl α-glucosides. Thus, anomalies in that mutant *H. pylori* may have perturbed the immune response.

Among three major *H. pylori* cholesteryl α-glucosides, αCPG was identified as the most potent antigen for *i*NKT cells based on an *in vitro* assay, even though the response toward αCPG was less potent than toward α-galactosylceramide. It has been shown that CD1d has two pockets at the α-galactosylceramide binding site and that the two acyl chains of the latter fit into these pockets [52]. It is possible that two acyl chains of αCPG similarly fit into these pockets. When these different cholesteryl α-glucosides are presented from DCs, αCPG (monoacyl) is the best antigen *in vivo*. Therefore, *in vivo* one acyl chain and cholesterol with a side chain may be the optimal antigen for *i*NKT interaction with the CD1d pocket.

*i*NKT cells reportedly produce cytokines that stimulate different immune cells [19]. Under the experimental conditions used, an increase in mononuclear cells was not observed 10 days after infection [42]. *i*NKT cells constitute less than 3% of all T lymphocytes in many tissues [53]. Because of their low abundance, an increase in *i*NKT cells was not observed in either WT or Jα18^−/−^ mice. It has been reported that *H. pylori* infection leads to Th1 cell activation [43]. However, Th2 cell activation has not been well described [54]. The present work shows that 10 days after *H. pylori* inoculation, the Th2 cell response is as robust as the Th1 cell response, and that those responses largely depend on *i*NKT cells. Since Th2 cells play a role in the tolerogenic response [44], Th2 cell activation by *H. pylori* may promote long-lasting attenuation of the immune response, which might underlie the chronic nature of *H. pylori* infections. In our present study, we did not detect an increase in Tregs ten days after infection, while a recent study reports that Tregs increase during influenza virus infection in the presence of *H. pylori* or αCAG [55]. Our findings suggest that the increase in Th2 cells induced by excess cholesteryl α-glucosides may promote a tolerogenic effect following *H. pylori* infection through *i*NKT cell activation.

The inflammatory response toward *H. pylori* infection exemplifies an inflammatory response that leads to cancer [50], [56]. The novel function of *H. pylori* glycans that we report here significantly extends our previous understanding of the roles of glycosylation in pathogenesis [57]. Future studies should determine whether αCgT inhibition constitutes an alternative treatment for *H. pylori*-induced inflammation and cancer.

## Materials and Methods

### *H. pylori* strain and bacterial culture

The standard *H. pylori* strain 26695 (ATCC700392) was purchased from American Type Culture Collection (ATCC, Manassas, VA), and routinely grown on Tripticase Soy agar with 5% sheep blood (TSA II) (Becton Dickinson, Franklin Lakes, NJ) for 2 to 3 days at 35°C in 12% CO_2_. Bacteria were precultured in Brucella broth (Becton Dickinson) supplemented with 10% fetal bovine serum (FBS) (HyClone, Logan, UT). Subsequently, bacteria was diluted to 4×10^7^ cells/ml and cultured in brain heart infusion (Becton Dickinson) supplemented with 0.2% yeast extract (Becton Dickinson) and 10% FBS (BHI/YE/FBS). Bacteria cultured in BHI/YE/FBS were used for all experiments except for generation of recombinant αCgT mutants. For targeting of the αCgT gene, bacteria on TSAII plates were used directly without liquid culture.

### Mice

C57BL/6 mice were purchased from the Jackson Laboratory. Jα18^−/−^ mice on a C57BL/6 background were generated by Dr. Masaru Taniguchi (Riken, Yokohama, Japan) [41]. All mice were housed in specific pathogen-free conditions. Animals were treated according to the guidelines of the National Institute of Health and the experiments were approved by the Institutional Animal Care and Use Committee of the Sanford-Burnham Medical Research Institute (PHS-Assurance number; A3053-01).

### Ethics statement

The experimental protocol and use of all human pathology specimens for research were approved by the Ethical Committee of Shinshu University School of Medicine (Matsumoto, Japan). The Ethical Committee also granted a waiver of informed consent to use *H. pylori* clinical isolates and the formalin-fixed and paraffin-embedded biopsy specimens retrieved from the pathology file of the Shinshu University Hospital, because the diagnostic use of the samples was completed before the study. Thus no risk to the patients involved was predicted. Samples were also coded to protect patient anonymity.

### Clinical isolates

Twenty-four gastric biopsy specimens from Japanese patients were obtained by endoscopic examination at Shinshu University Hospital, Matsumoto, Japan. The patients consisted of 7 male and 17 female (ranging in age from 12 to 83 years; average 49.7 years), and only *H. pylori*-positive patients were evaluated. For histological assessment of chronic gastritis, at least 5 biopsies were taken; biopsy specimens were fixed in phosphate-buffered 10% formalin (WAKO, Osaka, Japan), embedded in paraffin, and cut into 5-µm-thick sections. Pathological diagnosis was evaluated based on the updated Sydney System.

Clinical bacterial stocks from each specimen were stored at −80°C. *H. pylori* were cultivated at 35°C under microaerophilic conditions. *H. pylori*-selective agar plates were utilized (Eiken, Tokyo, Japan), and single colonies were incubated on TSAII plates (Becton Dickinson) for isolation of genomic DNA. Similarly, genomic DNA was isolated from 5 clinical isolates from India, 10 from Sweden, 3 from Germany, and 5 from Spain, all of which were stored in Umeå University, Umeå, Sweden [58]. Genotyping for *cagA* and *vacA* was conducted using published PCR primers [59]. The experimental protocol was approved by the Ethics Committee of Shinshu University School of Medicine and Umeå University.

### Generation of αCgT mutants by homologous recombination

The strategy used to disrupt the αCgT gene and replace mutated αCgT from clinical isolates is shown in Figure S2 and File S1. Briefly, αCgT^Δ^ were bacteria deficient in αCgT, and αCgT^high^ and αCgT^low^ harbored high and low activities, respectively, of αCgT. αCgT^cont^ was a control clone.

### Preparation of lysates from WT *H. pylori* and αCgT mutants

Precultured *H. pylori* (WT and recombinant αCgT mutants) were washed with PBS twice. 1×10^8^ CFU of bacteria were resuspended in 200 µl PBS, sonicated 10 times each for 10 sec at 30 sec intervals, and then stored at −80°C until use.

### *In vitro* response to *i*NKT hybridoma cells

Lysates from recombinant αCgT^Δ^ or WT *H. pylori* (at 1×10^7^ or 2×10^6^ CFU/well, respectively), or 1 µg/well of synthetic compounds were incubated for 24 hours in 96-well microplates coated with 10 µg/ml mouse CD1d-tetramer, according to published methods [60]. For controls, 6 ng/well of α-galactosylceramide and/or 1×10^7 ^CFU/well *S. yanoikuyae* lysate were used [27]. After washing wells with PBS, 1×10^5^ of mouse *i*NKT hybridoma cells (clone 1.2 or 1.4) were cultured for 16 hours, and then IL-2 secreted into supernatants was measured by a sandwich ELISA (BD Pharmingen, La Jolla, CA). Synthetic glycolipids were initially dissolved in dimethyl sulfoxide (DMSO) and then prepared using a series of 10-fold dilutions with assay medium prior to the assay. In parallel, the glycolipids were incorporated in liposomes as described previously [61] and assayed after 20-fold dilution.

### *In vivo i*NKT cell activation

Mouse dendritic cells were prepared by culturing bone marrow cells in media containing 10 ng/ml mouse recombinant GM-CSF (Kyowa-Hakko-Kirin, Tokyo, Japan) for 7 days. 1×10^6^ DCs were then incubated with 50 µg/ml of synthetic cholesteryl α-glucosides, 100 ng/ml of α-galactosylceramide, or 500 ng/ml of BbGL-IIc (a glycolipid derived from *B. Burgdorferi*) for 24 hours. After washing cells with PBS, 5×10^5^ of glycolipid-pulsed DCs were intravenously injected into C57BL/6 WT mice. Liver mononuclear cells were collected 16 hours later and analyzed directly for IFN-γ and TNF-α levels by FACS Calibur (BD Bioscience) in *i*NKT cells. Intracellular cytokine staining of α-galactosylceramide-CD1d tetramer-positive cells was carried out according to a published protocol [60].

### Short-term *H. pylori* infection assay

C57BL/6 WT or Jα18^−/−^ mice were fasted overnight and orogastrically inoculated with 3×10^8^ CFU *H. pylori* in BHI/YE/FBS by a gastric intubation tube three times at one-day intervals. Mice were maintained on a fasting regime for an additional 4 hours after each infection. Control mice were administered media only.

Mice were sacrificed at post-infection day 10 after the third bacterial infection, and the stomach was cut into along the greater curvature, washed with diethylpyrocarbonate (DEPC)-treated PBS, and divided into two pieces along the lesser curvature. For each stomach, one piece was used for measuring wet weight and then placed in 1 ml PBS for quantification of bacterial colonies. The other piece was immediately frozen on dry ice and stored at −80°C for total RNA isolation. RNA extraction and RT-PCR analysis was performed as described in File S1.

### Quantification of *H. pylori* colonies from mouse stomach

Stomach tissue prepared as described above was homogenized in 1 ml of PBS three times. Homogenates at 1∶5, 1∶10, 1∶20, and 1∶40 dilutions were prepared in PBS, and then 50 µl of each dilution was put onto an *H. pylori* selective-agar plate containing 30 µg/ml kanamycin in duplicate. Plates were incubated at 35°C for 5 days under microaerophilic conditions, and colonies were evaluated as CFU/gram of stomach tissue.

### Statistical analysis

Statistical analysis was carried out using Prism 5 (GraphPad Software, Inc., La Jolla, CA) and evaluated by an unpaired *t*-test. *P* values of <0.05 were considered statistically significant. Correlation coefficients as described by *r* values were analyzed by calculating Spearman's rank correlation coefficient.

Other experimental procedures are described in File S1.

## Supporting Information

Figure S1 **Homologous recombination strategy used to generate** ***H. pylori*** **harboring αCgT from different** ***H. pylori*** **isolates, related to** **Figure 3** **.** For all manipulations, the kanamycin resistance gene served as a selectable marker. (A) Construction of αCgT-deficient *H. pylori* (αCgT^Δ^). (B) Replacement by homologous recombination of the *H. pylori* 26695 αCgT gene with entire sequences of mutant αCgT derived from clinical isolates, generating *H. pylori* αCgT^high^ and αCgT^low^. αCgT^cont^ harboring no αCgT mutations was created as a control. The efficiency of homologous recombination was improved over the previous report [14].
(TIF)

Figure S2 **Evaluation of progression of stomach anomalies including peptic ulcer and inflammation, related to** **Figure 4** **.** (A) Schematic representation of 5 biopsy sites evaluated using the updated Sydney System. Assessed were the lesser curvature of the antrum (A1), the greater curvature of the antrum (A2), the smaller curvature of the angle (IA), the lesser curvature of the middle body (B1), and the greater curvature of the upper body (B2). (B) Histological criteria were evaluated as normal (0), mild (1), moderate (2), and marked (3) degrees of (from left to right) *H. pylori* infection, infiltration of neutrophils and mononuclear cells, atrophy (antrum and corpus), and intestinal metaplasia in five stomach regions of the stomach (left). Scores of each for sample #1 are shown.
(TIF)

Figure S3 **Macrophage and dendritic cell responses to different** ***H. pylori*** **clones.** (A) THP-1 cells were differentiated by adding phorbol 12-myristate 13-acetate, and 72 hours later different forms of *H. pylori* (4×10^8^ CFU/ml) expressing αCgT^high^, αCgT^low^, αCgT^cont^, αCgT^Δ^, or WT *H. pylori* 26695 were added to 2×10^5^ differentiated THP-1 cells followed by washing. After 20 hours phagocytosis was evaluated by counting remaining *H. pylori*. Two *H. pylori* clones for each mutant were analyzed. Means ± S. E. M are shown. (B) CD14-positive cells isolated from human peripheral blood were incubated with IL-4 and GM-CSF for 6 days and those differentiated dendritic cells were then incubated with *H. pylori* lysates at a MOI (*H. pylori*/dendritic cells) of 5 for 48 hours at 37°C. DC maturation/activation was then determined by FACS analysis. CD11c was used to gate mature DCs, and CD86 and HLA-DR expression was determined as markers of antigen-presentation. Expression of CD40, a differentiation marker for DC cells, was also measured. Expression on immature DC cells before pulse is shown in blue. The results represent one of two repeated experiments.
(TIF)

Figure S4 **Structures of αCGL, αCAG, and αCPG (monoacyl), related to** **Figures 5** **and S6.** Structures of αCGL, βCGL, αCAG, αCPG, αCPG (monoacyl), and α-galactosylceramide are shown. α-linkage is included in all structures except βCGL. αCPG and αCPG (monoacyl) possess fatty acid chain(s) as does α-galactosylceramide.
(TIF)

Figure S5 ***i*** **NKT cell activation by synthetic cholesterol α-glycosides and their binding to CD1d, as assessed by isoelectrofocusing.** (A) *i*NKT cell activity toward different cholesterol α-glycosides *in vitro*. 1 µg/well of synthetic αCGL, βCGL, αCAG, αCPG were presented in liposome form or dissolved initially in DMSO. α-galactosylceramide (6 ng/well) (αGC) and 1×10^7^ CFU/well of a lysate from *S. yanoikuyae* served as positive controls. IL-2 was measured as in Fig. 5A and Fig. S5. As indicated, αCPG is a much more potent antigen than αCGL *in vitro*. Data represent means ± S. E.M. (B) Binding of αCPG (monoacyl) to CD1d assessed by isoelectrofocusing. Incubation of CD1d with indicated lipids resulted in the appearance of a band with altered mobility (arrow) only in presence of αCPG (monoacyl). The extent of that shift is comparable to the one observed for the negatively charged sulfatide. That shift is interpreted as resulting from addition of negatively charged phosphate present on lipid of the protein-lipid complex.
(TIF)

Figure S6 **Photomicrographs of stomachs from WT and Jα18^−/−^ mice, related to** **Figure 6** **and S9.** Stomachs (fundus and antrum) from WT (A) and Jα18^−/−^ (B) mice were collected 10 days after the last infection with *H. pylori*. Hematoxylin and eosin staining was used. Bar, 100 µm.
(TIF)

Figure S7 **Expression of human Vα24Jα18 in paraffin-embedded gastric tissue specimens.** Q-PCR analysis of Vα24Jα18 transcript levels in formalin-fixed, paraffin-embedded gastric biopsy specimens relative to levels seen in control human stomach (values set to 1.0). Shown are relative expression of Vα24Jα18 mRNA in 7 patients (A) and median values (25–75 percentile) (B). Vα24Jα18 mRNA expression was not detected in specimens from the remaining 9 patients. Total RNA derived from normal human stomach (purchased from Clontech) served as a reference control. The transcript of αCgT was not detected by RT-PCR. Anomalous levels seen in patient #16 sample could be due to decreased *H. pylori*, which was eradicated by antibiotic treatment before biopsies were taken.
(TIF)

Figure S8 **Expression of cytokines and immune cell markers in** ***H. pylori*** **-infected stomach tissues 10 days after infection, related to** **Figure 6** **.** Transcript levels of IL-2 (a Th1 cytokine, A), IL-5 and IL-6, (Th2 cytokines, B and C), IL-10 (a regulatory cytokine, D) IL-17A and IL-22 (Th17 cytokines, E and H), RORγt (a Th17 cell marker, G), and LTβ (F) were determined by RT-PCR. For each experiment, four WT or Jα18^−/−^ mice were infected with *H. pylori*. Stomach samples shown in Figure 6 in the text were used. Statistical significance was evaluated using an unpaired *t*-test (*, *P*<0.05). Mean ± S. D. are shown.
(TIF)

File S1 **Supporting Information.** **Table S1. Primers used to amplify fragments of the 5′ arm, the** ***KanR*** **gene, 〈CgT and the 3′ arm in targeting vectors. Table S2. Primers used for RT-PCR of** ***H. pylori*** **-infected mouse stomach tissues.**
(PDF)
