# Supplementary figures and images for: Helicobacter pylori Cholesteryl α-Glucosides Contribute to Its Pathogenicity and Immune Response by Natural Killer T Cells
Source: PLoS One. 2013 Dec 2;8(12):e78191. doi: 10.1371/journal.pone.0078191 (PMC3846475; doi:10.1371/journal.pone.0078191)

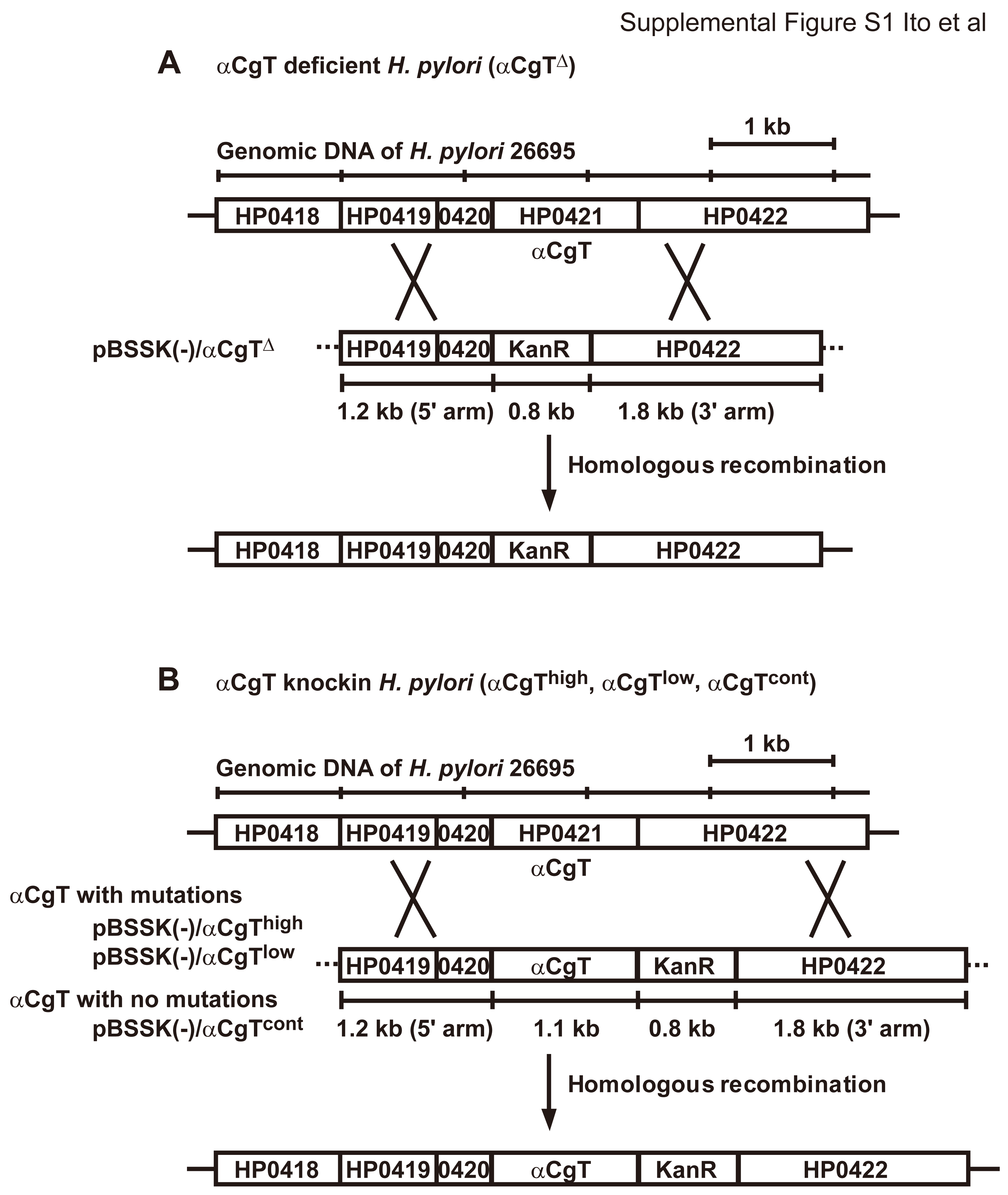

Supplement: Figure S1 — Homologous recombination strategy used to generate H. pylori harboring αCgT from different H. pylori isolates, related to Figure 3 . For all manipulations, the kanamycin resistance gene served as a selectable marker. (A) Construction of αCgT-deficient H. pylori (αCgTΔ). (B) Replacement by homologous recombination of the H. pylori 26695 αCgT gene with entire sequences of mutant αCgT derived from clinical isolates, generating H. pylori αCgThigh and αCgTlow. αCgTcont harboring no αCgT mutations was created as a control. The efficiency of homologous recombination was improved over the previous report [14]. (TIF) [file pone.0078191.s001.tif]

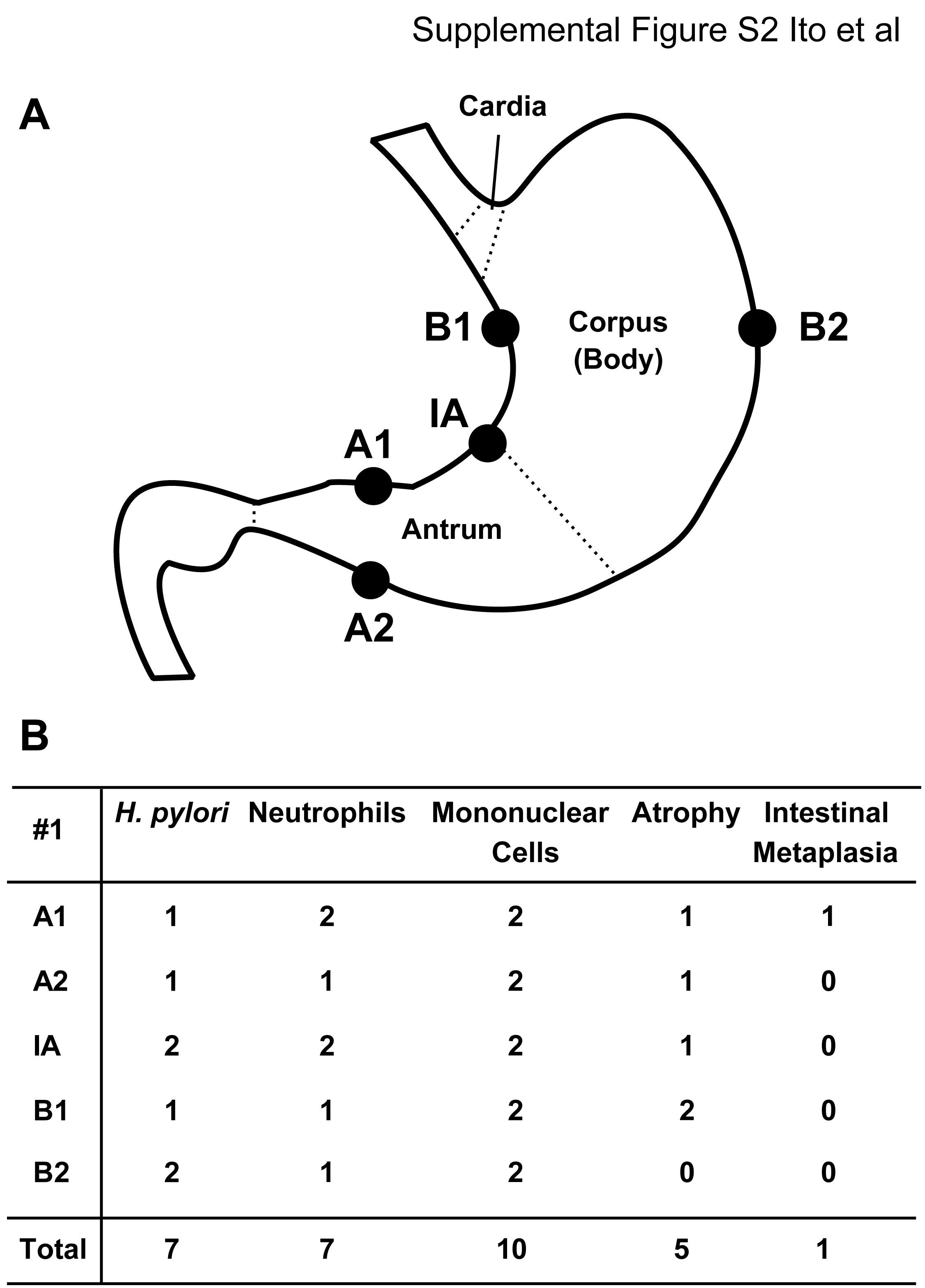

Supplement: Figure S2 — Evaluation of progression of stomach anomalies including peptic ulcer and inflammation, related to Figure 4 . (A) Schematic representation of 5 biopsy sites evaluated using the updated Sydney System. Assessed were the lesser curvature of the antrum (A1), the greater curvature of the antrum (A2), the smaller curvature of the angle (IA), the lesser curvature of the middle body (B1), and the greater curvature of the upper body (B2). (B) Histological criteria were evaluated as normal (0), mild (1), moderate (2), and marked (3) degrees of (from left to right) H. pylori infection, infiltration of neutrophils and mononuclear cells, atrophy (antrum and corpus), and intestinal metaplasia in five stomach regions of the stomach (left). Scores of each for sample #1 are shown. (TIF) [file pone.0078191.s002.tif]

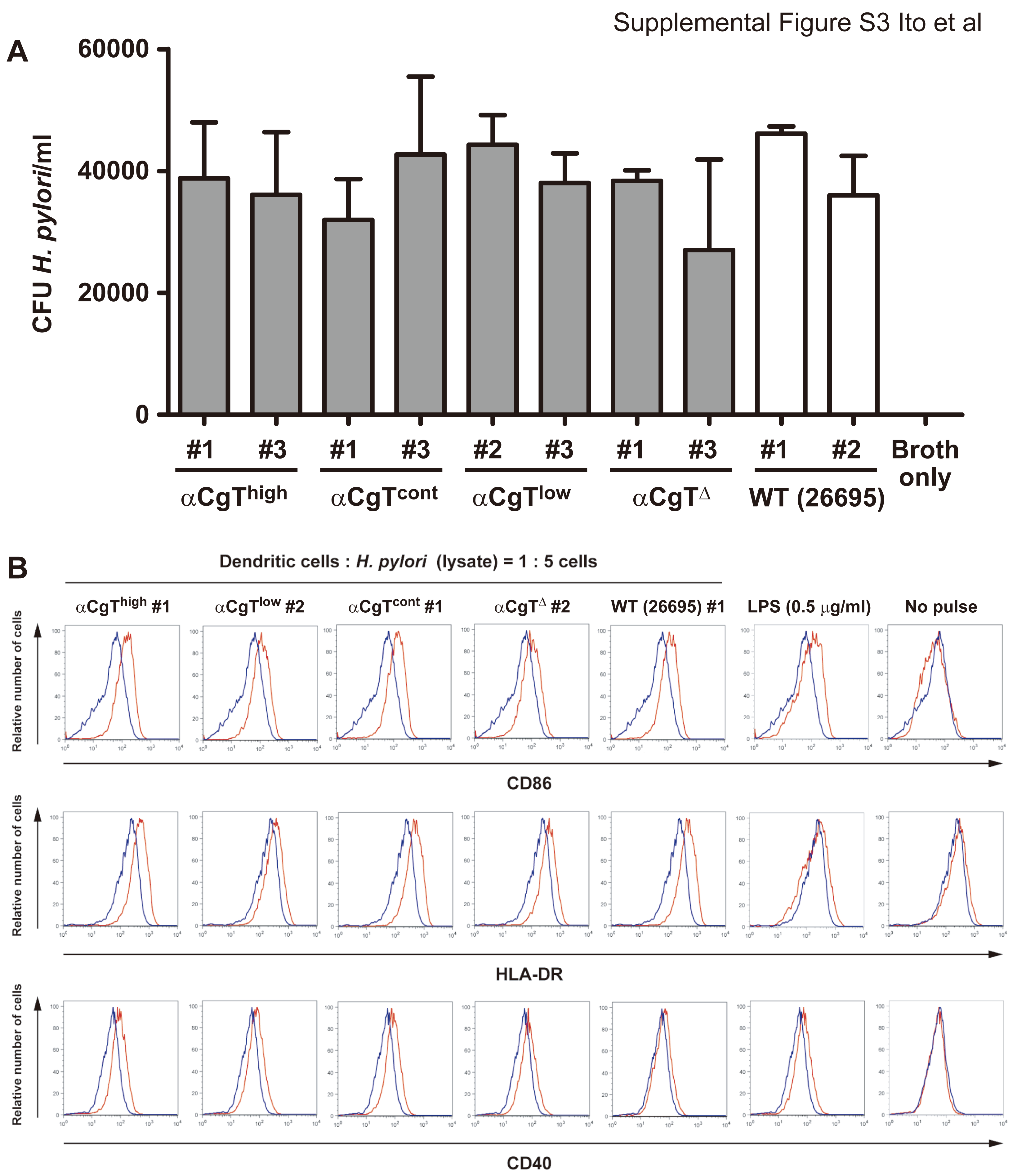

Supplement: Figure S3 — Macrophage and dendritic cell responses to different H. pylori clones. (A) THP-1 cells were differentiated by adding phorbol 12-myristate 13-acetate, and 72 hours later different forms of H. pylori (4×10^8^ CFU/ml) expressing αCgThigh, αCgTlow, αCgTcont, αCgTΔ, or WT H. pylori 26695 were added to 2×10^5^ differentiated THP-1 cells followed by washing. After 20 hours phagocytosis was evaluated by counting remaining H. pylori. Two H. pylori clones for each mutant were analyzed. Means ± S. E. M are shown. (B) CD14-positive cells isolated from human peripheral blood were incubated with IL-4 and GM-CSF for 6 days and those differentiated dendritic cells were then incubated with H. pylori lysates at a MOI (H. pylori/dendritic cells) of 5 for 48 hours at 37°C. DC maturation/activation was then determined by FACS analysis. CD11c was used to gate mature DCs, and CD86 and HLA-DR expression was determined as markers of antigen-presentation. Expression of CD40, a differentiation marker for DC cells, was also measured. Expression on immature DC cells before pulse is shown in blue. The results represent one of two repeated experiments. (TIF) [file pone.0078191.s003.tif]

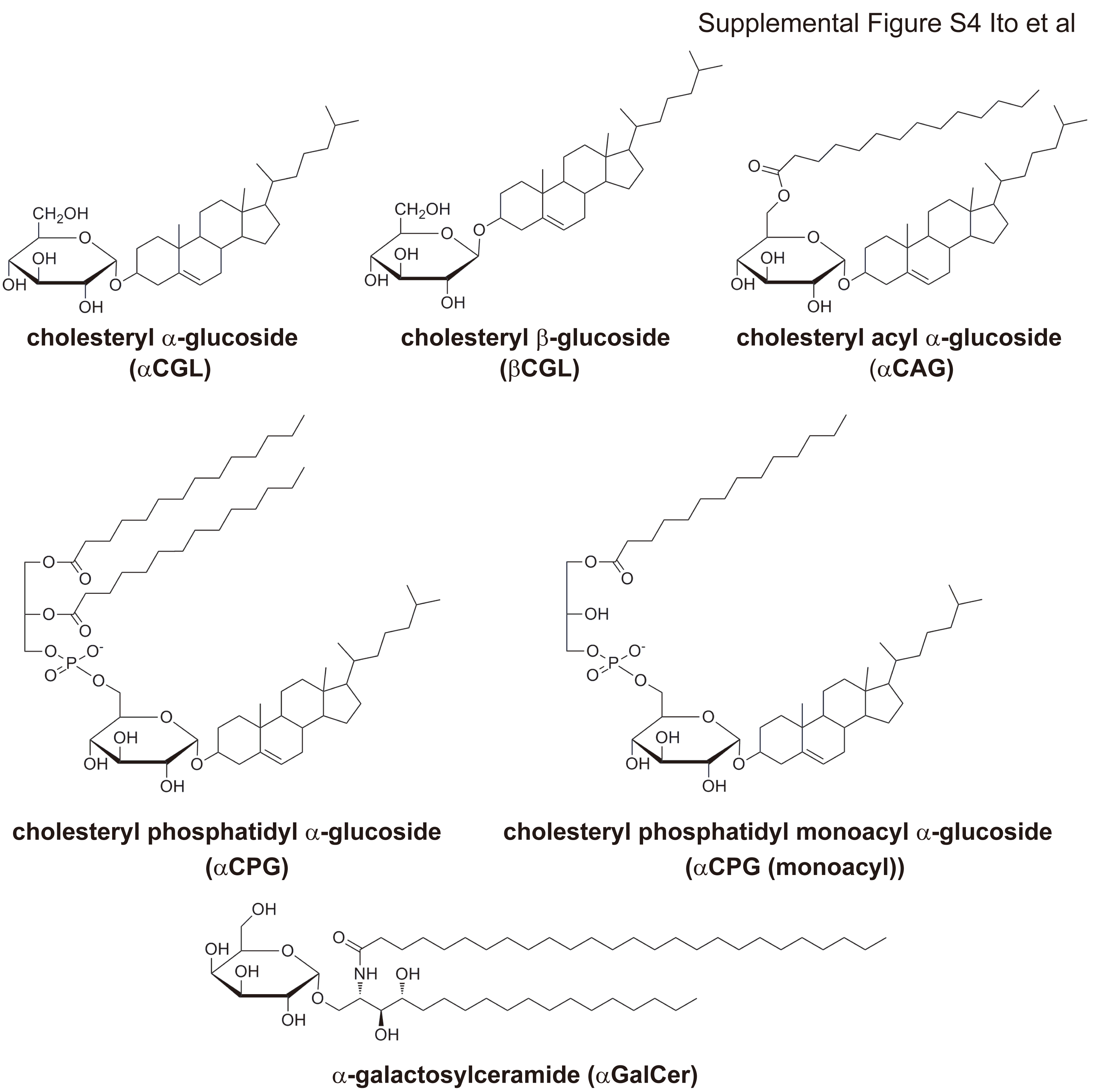

Supplement: Figure S4 — Structures of αCGL, αCAG, and αCPG (monoacyl), related to Figures 5 and S6. Structures of αCGL, βCGL, αCAG, αCPG, αCPG (monoacyl), and α-galactosylceramide are shown. α-linkage is included in all structures except βCGL. αCPG and αCPG (monoacyl) possess fatty acid chain(s) as does α-galactosylceramide. (TIF) [file pone.0078191.s004.tif]

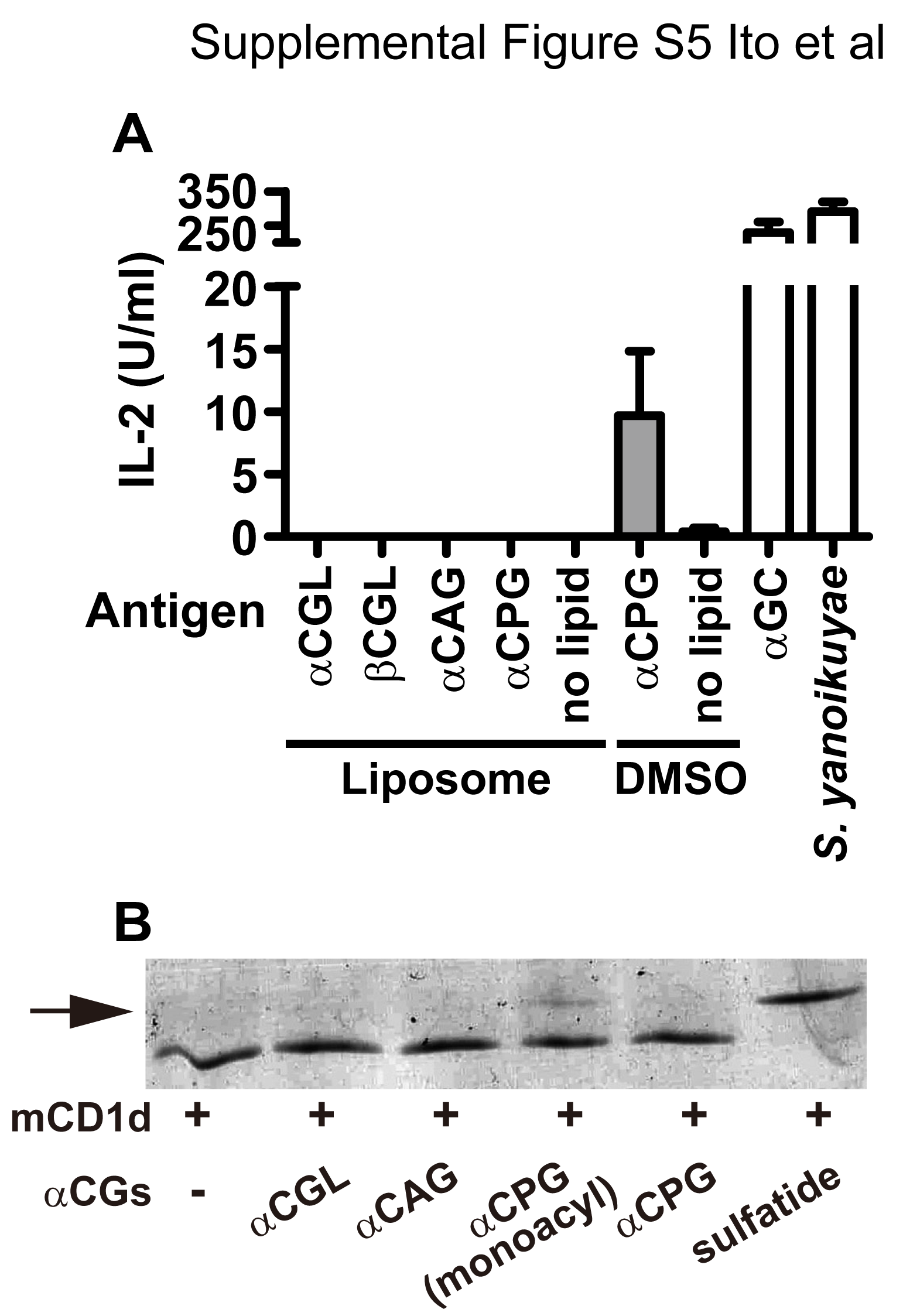

Supplement: Figure S5 — i NKT cell activation by synthetic cholesterol α-glycosides and their binding to CD1d, as assessed by isoelectrofocusing. (A) iNKT cell activity toward different cholesterol α-glycosides in vitro. 1 µg/well of synthetic αCGL, βCGL, αCAG, αCPG were presented in liposome form or dissolved initially in DMSO. α-galactosylceramide (6 ng/well) (αGC) and 1×10^7^ CFU/well of a lysate from S. yanoikuyae served as positive controls. IL-2 was measured as in Fig. 5A and Fig. S5. As indicated, αCPG is a much more potent antigen than αCGL in vitro. Data represent means ± S. E.M. (B) Binding of αCPG (monoacyl) to CD1d assessed by isoelectrofocusing. Incubation of CD1d with indicated lipids resulted in the appearance of a band with altered mobility (arrow) only in presence of αCPG (monoacyl). The extent of that shift is comparable to the one observed for the negatively charged sulfatide. That shift is interpreted as resulting from addition of negatively charged phosphate present on lipid of the protein-lipid complex. (TIF) [file pone.0078191.s005.tif]

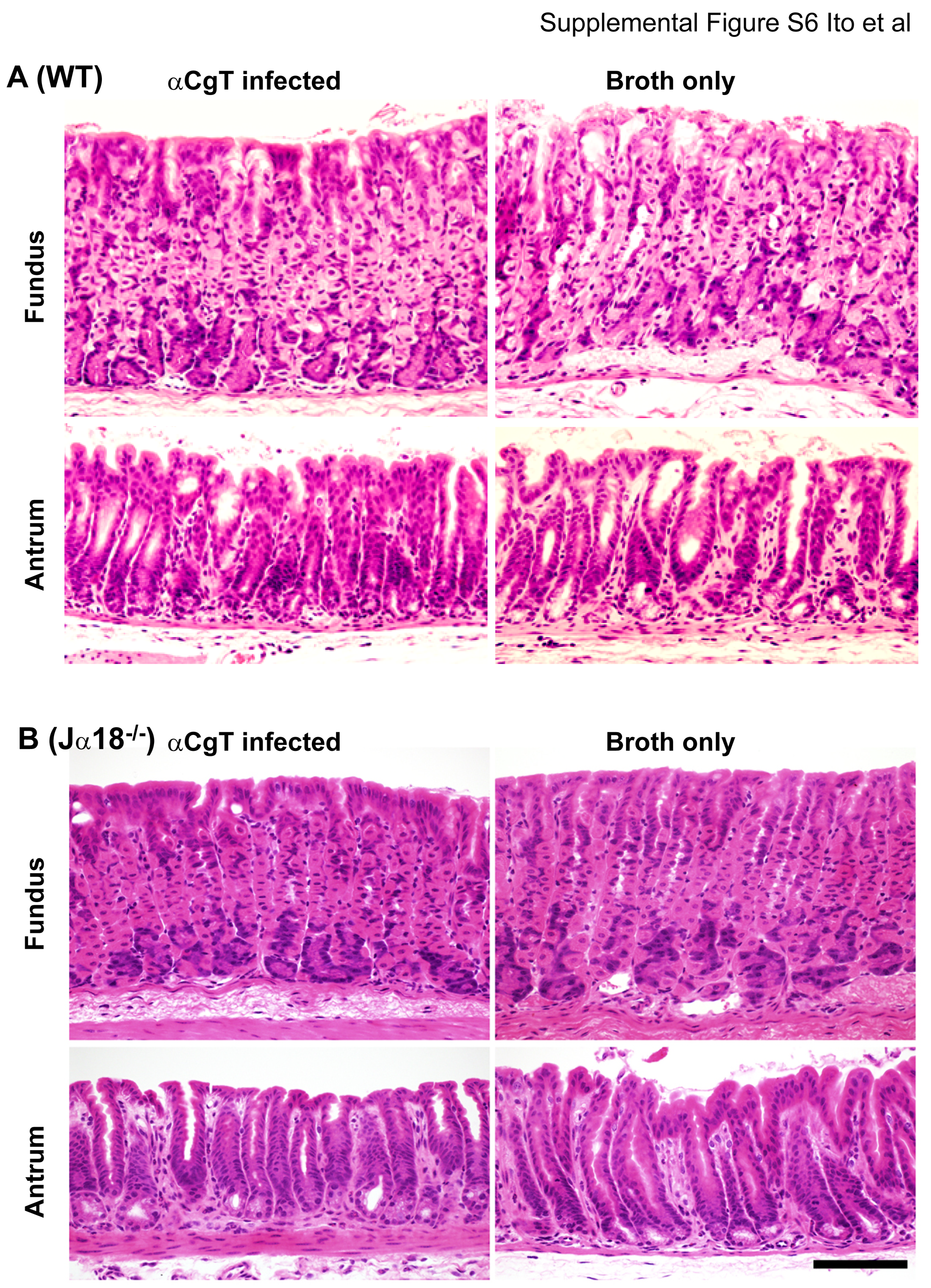

Supplement: Figure S6 — Photomicrographs of stomachs from WT and Jα18−/− mice, related to Figure 6 and S9. Stomachs (fundus and antrum) from WT (A) and Jα18−/− (B) mice were collected 10 days after the last infection with H. pylori. Hematoxylin and eosin staining was used. Bar, 100 µm. (TIF) [file pone.0078191.s006.tif]

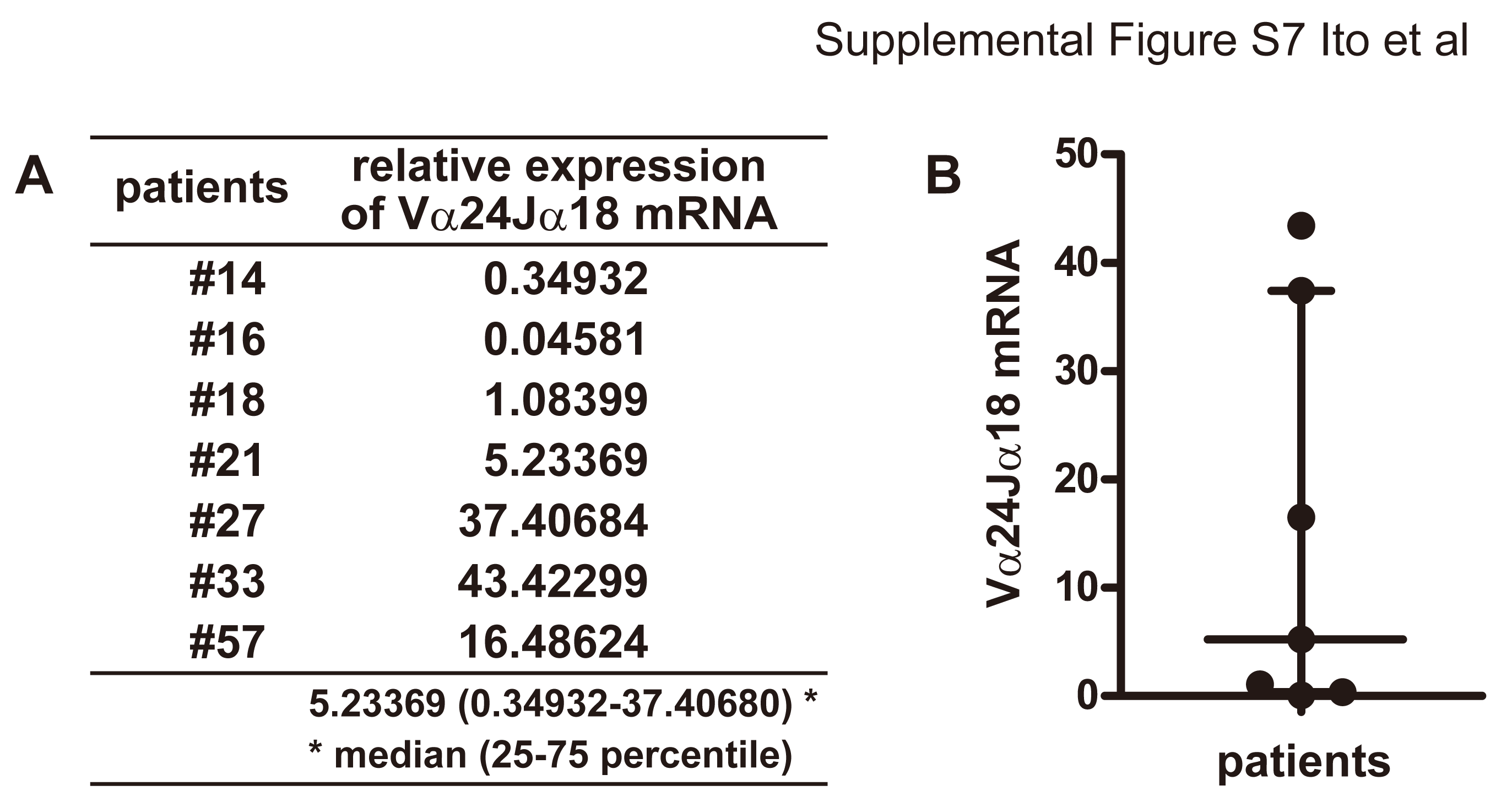

Supplement: Figure S7 — Expression of human Vα24Jα18 in paraffin-embedded gastric tissue specimens. Q-PCR analysis of Vα24Jα18 transcript levels in formalin-fixed, paraffin-embedded gastric biopsy specimens relative to levels seen in control human stomach (values set to 1.0). Shown are relative expression of Vα24Jα18 mRNA in 7 patients (A) and median values (25–75 percentile) (B). Vα24Jα18 mRNA expression was not detected in specimens from the remaining 9 patients. Total RNA derived from normal human stomach (purchased from Clontech) served as a reference control. The transcript of αCgT was not detected by RT-PCR. Anomalous levels seen in patient #16 sample could be due to decreased H. pylori, which was eradicated by antibiotic treatment before biopsies were taken. (TIF) [file pone.0078191.s007.tif]

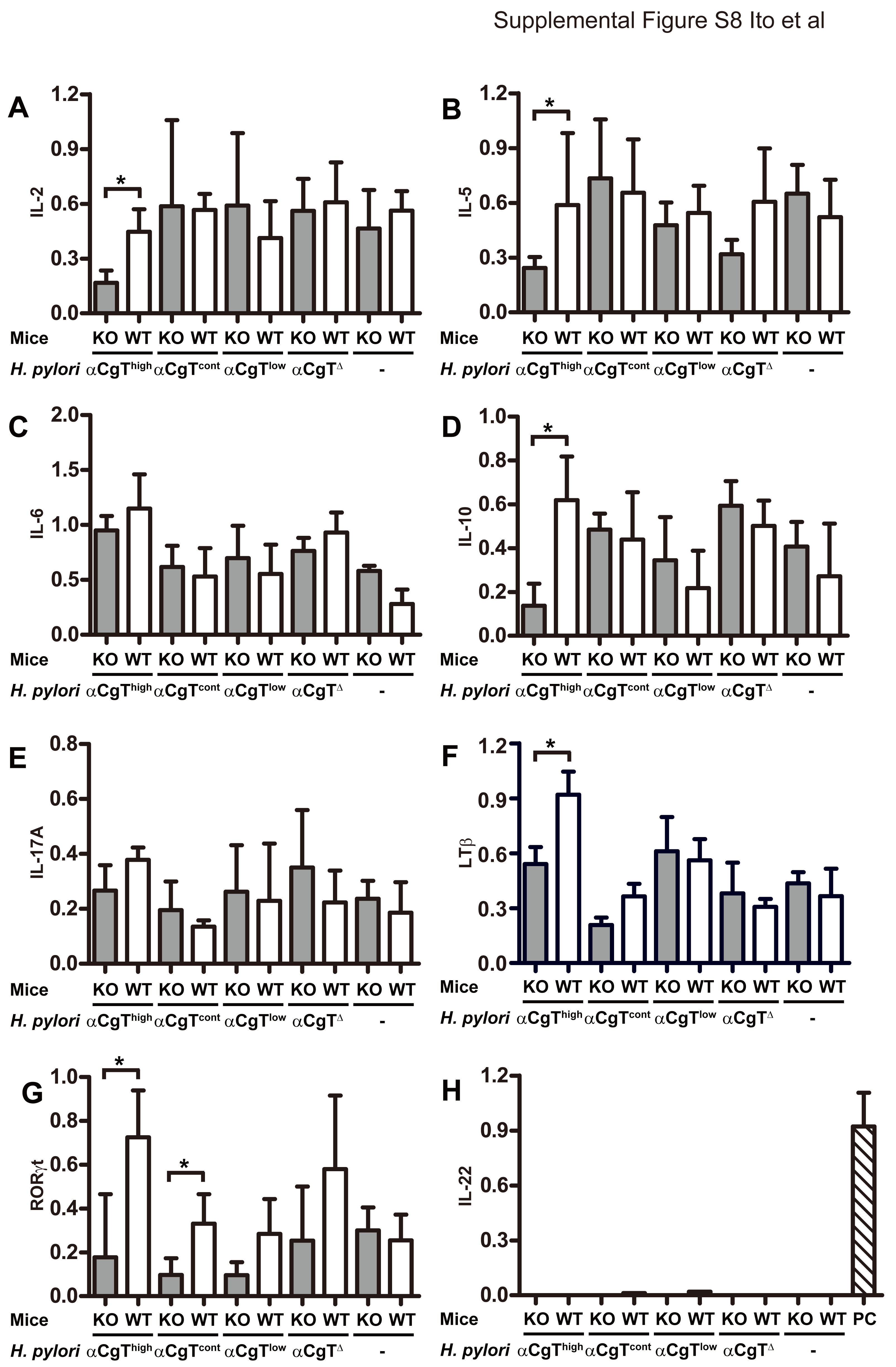

Supplement: Figure S8 — Expression of cytokines and immune cell markers in H. pylori -infected stomach tissues 10 days after infection, related to Figure 6 . Transcript levels of IL-2 (a Th1 cytokine, A), IL-5 and IL-6, (Th2 cytokines, B and C), IL-10 (a regulatory cytokine, D) IL-17A and IL-22 (Th17 cytokines, E and H), RORγt (a Th17 cell marker, G), and LTβ (F) were determined by RT-PCR. For each experiment, four WT or Jα18−/− mice were infected with H. pylori. Stomach samples shown in Figure 6 in the text were used. Statistical significance was evaluated using an unpaired t-test (*, P<0.05). Mean ± S. D. are shown. (TIF) [file pone.0078191.s008.tif]
