# Supplementary material for: Helicobacter pylori Cholesteryl α-Glucosides Contribute to Its Pathogenicity and Immune Response by Natural Killer T Cells
Source: PLoS One. 2013 Dec 2;8(12):e78191. doi: 10.1371/journal.pone.0078191 (PMC3846475; doi:10.1371/journal.pone.0078191)
Supplement: File S1 — Supporting Information. Table S1. Primers used to amplify fragments of the 5′ arm, the KanR gene, 〈CgT and the 3′ arm in targeting vectors. Table S2. Primers used for RT-PCR of H. pylori -infected mouse stomach tissues. (PDF) [file pone.0078191.s009.pdf]

SUPPLEMENTAL INFORMATION

SUPPLEMENTAL TABLE

Table S1. Primers used to amplify fragments of the 5' arm, the *KanR* gene, αCgT and the 3' arm in targeting vectors.

Primers	Sequences (5' to 3')[a]	Site	Products (bp)
5' arm sense	CGGGGTACCATCATGCTCATTTGTAACGATAAATC	*Kpn*I	1222[b]
5' arm antisense	ATCCTTCTCGAGTTAACTACTCTTCTTTAAAATTG	*Xho*I	
αCgT sense	ATCCTTCTCGAGATGGTTATTGTTTTAGTCGTGG 3'	*Xho*I	1170[c]
αCgT antisense	AAAACTGCAGTTATGATAAGGTTTTAAAGAGATGG	*Pst*I	
KanR sense	AAAACTGCAGTGAAAGGATAAAAAATGAGCCATATTCAACGG	*Pst*I	816[d]
KanR antisense	CGCGGATCCTTAGAAAAACTCATCGAGCATC 3'	*Bam*HI	
3' arm sense	CGCGGATCCTGAAAGGATAAAAAATGCAAGAAG 3'	*Bam*HI	1879[e]
3' arm antisense	GCGAGCTCTAGCGATTAAAGCCGTTTAAATC 3'	*Sac*I	

(a) Restriction sites are underlined.

The thermal conditions were as below:

(b) 98°C for 30 sec; 35 cycles of 98°C for 10 sec, 55°C for 5 sec, 72°C for 2 min; followed by 72°C for 3 min.

(c) 98°C for 30 sec; 35 cycles of 98°C for 10 sec, 58°C for 5 sec, 72°C for 1 min; followed by 72°C for 3 min.

(d) 98°C for 30 sec; 30 cycles of 98°C for 10 sec, 55°C for 5 sec, 72°C for 1 min; followed by 72°C for 3 min.

(e) 98°C for 30 sec; 40 cycles of 98°C for 10 sec, 58°C for 30 sec, 72°C for 1 min; followed by 72°C for 3 min.

Table S2. Primers used for RT-PCR of *H. pylori*-infected mouse stomach tissues.

mRNA	Primers	Sequences (5' to 3')	Products (bp)	References
Vα14Jα18	sense	GACCCAAGTGGAGCAGAGTCCT	300[a]	[62]
	antisense	CAGCTCCAAAATGCAGCCTCCCTAA		
β-actin	sense	TGGAATCCTGTGGCATGCATGAAAC	348[b]	[63]
	antisense	TAAAACGCAGCTCAGTAACAGTCCG		
Foxp3	sense	TTCATGCATCAGCTCTCCAC	190[c]	[64]
	antisense	CTGGACACCCATTCCAGACT		

Other primer sequences, except for mouse cytokines, will be provided upon request.

(a) 95°C for 2 min; 39 cycles of 95°C for 30 sec, 62°C for 30 sec, 72°C for 30 sec; followed by 72°C for 3 min.

(b) 95°C for 2 min; 24 cycles of 95°C for 30 sec, 55°C for 30 sec, 72°C for 30 sec; followed by 72°C for 3 min.

(c) 95°C for 2 min; 40 cycles of 95°C for 30 sec, 57°C for 30 sec, 72°C for 30 sec; followed by 72°C for 3 min.

SUPPLEMENTAL METHODS

Detection of αCgT mutations from clinical isolates

Genomic DNA encoding αCgT from wild-type *H. pylori* 26695 and clinical isolates was amplified by PCR using primers containing *Nde*I (5') and *Xho*I (3') restriction sites. Purified PCR products were cloned into pTKNd6xH [14], resulting in pTKNd6xH/αCgT (WT) and pTKNd6xH/αCgT (samples #1-#24). All clones were sequenced for αCgT and, the sequence was compared to that of the standard strain.

Site-directed mutagenesis

Twenty amino acid substitutions in αCgT were detected from Japanese clinical isolates. *In vitro* site-directed mutagenesis was performed using a protocol established by Stratagene. pTKNd6xH/αCgT (WT) served as a template vector. For each mutation, oligonucleotide primers containing the mutant and complementary sequence were designed; primer sequences will be provided upon request. After digestion of parental DNA with 20 U of *Dpn*I (New England Biolabs), vector DNA containing the mutation was transformed into BL21 (Novagen) competent cells for repair and mutations were confirmed by DNA sequencing.

Generation of αCgT mutants by homologous recombination

The strategy used to disrupt the αCgT gene and replace mutated αCgT from clinical isolates is shown in Figure S2. Two flanking fragments, the 5' and 3' arms of αCgT from *H. pylori* 26695, and the kanamycin resistance gene (*kanR*) from the pET-28a(+) vector (Novagen, Gibbstown, NJ) were amplified using primers described in Table S1. All components flanked by

different restriction sites were cloned into pBluescript SK (-) (Stratagene, La, Jolla, CA), resulting in pBSSK(-)/αCgT$^{\Delta}$ deficient in αCgT gene.

For αCgT knock-in bacteria harboring high and low active αCgT, αCgT sequences from clinical isolates (#10, #17, and #52) were amplified using primers containing *Pst*I (5') and *Bam*HI (3') restriction sites and inserted between the 5' arm and the *kanR* into the pBSSK(-)/αCgT$^{\Delta}$ vector, as pBSSK(-)/αCgT^{high} and pBSSK(-)/αCgT^{low}. As a control, WT αCgT from *H. pylori* 26695 was similarly subcloned and constructed as pBSSK(-)/αCgT^{cont}.

H. pylori 26695 was electroporated by a method similar to that described by Segal [65]. Bacteria cultured on TSAII plates were harvested and washed with cold phosphate-buffered saline (PBS) twice. Cells were resuspended in cold electroporation buffer (9% glycerol/15% sucrose) at 2-5 x 10^8 cells/ml. Two micrograms of targeting vector (1 μg/μl in sterilized water) was mixed with a 100 μl cell suspension. The mixture was added to a 0.2 cm electroporation cuvette (Bio-Rad Laboratories, Hercules, CA) and subjected to a single-pulse of initial voltage 2.5 kV, 25 μF, and 400Ω in parallel. Bacteria was transferred onto a cold TSAII plate and incubated 24 hours at 35°C under 12% CO_2. Cells were then inoculated onto selective media with 30 μg/ml kanamycin and incubated 4 days to allow colony growth.

<u>αCgT enzyme assay</u>

Escherichia coli BL21 was transformed with pTKNd6xH/αCgT (WT), pTKNd6xH/αCgT (#1-#24), or pTKNd6xH/αCgT generated by site-directed mutagenesis. Histidine-tagged αCgT recombinant protein was purified using a HisTrap spin column (GE Healthcare, Little Chalfont, England). αCgT activity was assayed as previously described with

minor modifications [14]. Briefly, recombinant αCgT protein was incubated in 50 mM Hepes-NaOH (pH7.5) containing 20 μM (100,000 cpm) [^{3}H]UDP-glucose, 5 μM cholesterol, and 0.1% Triton X-100 for 10 min. The reaction was then stopped by adding HCl to 80 mM. Ten volumes of ethyl acetate were mixed and upper phase was collected for measurement of radioactivity after centrifugation.

<u>Modeling and refinement of method for αCgT</u>

Prior to homology modeling, a primary sequence alignment of αCgT in *H. pylori* 26695 (wild type) and phosphatidylinositol mannosyltransferase (PimA) was generated by Profile Multiple Alignment with Local Structure (PROMALS), a progressive multiple sequence alignment method [66]. An αCgT structural model was then created with the Alignment Mode of SWISS-MODEL using sequence alignment and the crystal structure of PimA (PDB code: 2GEJ) as template [67]. The structure of UDP-glucose (UDP-Glc) and its topology and parameter files were downloaded from HIC-Up server in the Hetero-compound Information Centre (Uppsala, Sweden) [68]. We manually replaced the 2GEJ's ligand, GDP-mannose (GDP-man), with UDP-Glc and rotated UDP-Glc's torsion angles to resemble GDP-man using the Crystallographic Object-Oriented Toolkit (COOT) program [69]. The resulting αCgT-UDP-Glc model was energy minimized with the Crystallography & NMR System (CNS), a program designed to provide a flexible multi-level hierachical approach [70].

Thin-layer chromatography (TLC)

Precultured wild-type or recombinant αCgT *H. pylori* was washed with 5 ml PBS three times, and then glycolipid fractions were extracted from 2 x 10^8 bacterial cells using chloroform/methanol (2:1=v/v) at room temperature for 1 hour. The organic phase was centrifuged at 3000 x g for 10 min and dried under a nitrogen stream. The residue was dissolved in 10 μl of chloroform/methanol (2:1=v/v) and subjected to TLC using chloroform/methanol/water (65/25/4=v/v/v). Separated glycolipids were visualized using orcinol-containing sulfuric acid buffer at 95°C.

***H. pylori* growth assay**

After preculturing, *H. pylori* 26695 (WT) and recombinant αCgT mutants were diluted to 1 x 10^7 cells/ml with BHI/YE/FBS. Bacteria in a 96-well plate was cultured at 35°C in 12% CO_2, and bacterial growth was measured by monitoring the OD at 600 nm each day for 4 days.

Scanning electron microscopy

Precultured *H. pylori* was fixed in 2.5% glutaraldehyde at 4°C overnight. Bacteria were washed three times in 0.1 M phosphate buffer (PB), pH 7.2, and then refixed in PB containing 1% osmium on ice for 1 hour, followed by serial dehydrations in ethanol. After centrifugation, bacteria were rinsed in t-butyl alcohol and placed into aluminum foil containers 5 mm in diameter. Containers were completely submerged in t-butyl alcohol for 10 min, followed by three washes with t-butyl alcohol. Samples were gradually frozen at –20°C and then lyophilized using a JFD-310 machine (JEOL, Tokyo, Japan). Containers were mounted on a copper plate and coated with osmium using osmium plasma coater OPC40 (Filgen, Nagoya, Japan). Micrographs

were obtained using a scanning electron microscope (JSM-6000F (JEOL)) at 15 kV accelerating voltage.

Phagocytosis assay

The human monocyte cell line THP-1 was differentiated by adding 20 nM phorbol 12-myristate 13-acetate (Sigma, St Lois, MO) in PRMI1640 (Mediatech Inc, Manassas, VA) supplemented with 2 mM L-glutamine and 10% FBS for 72 hours at 37°C in 5% CO_2. 4 x 10^8 CFU/ml of precultured *H. pylori* was dispersed with 2 x 10^5 macrophage-like differentiated cells at a multiplicity of infection (MOI) of 100 bacteria/macrophage. Phagocytosis was synchronized by centrifugation at 100 g for 2 min at RT and then allowed to proceed 60 min. Non-adherent bacteria were removed by washing with phosphate-buffered saline (PBS) twice and then incubated with RPMI1640 supplemented with 100 μg/ml gentamicin (Sigma) for 1 hour at 37°C. Macrophages were rinsed with PBS three times and maintained in antibiotic-free culture medium for 20 hours at 37°C. The macrophages were washed and lysed in 100 μl of 0.1% saponin in PBS, and serial dilutions of each sample were plated onto TSA II plates in duplicate and incubated for four days at 35°C under microaerophilic conditions to determine the number of CFU of *H. pylori.*

Human monocyte isolation

Peripheral blood mononuclear cells (PBMC) were obtained from venous blood of healthy adult volunteers not infected with *H. pylori*, using Ficoll-Hypaque Plus (GE Healthcare) with LeucoSep (Greiner Bio-One, Frickenhausen, Germany), according to the manufacturer's instructions.

Determination of human dendritic cell maturation

CD14-positive monocytes were isolated from PBMC by MACS with CD14 MicroBeads (Miltenyi Biotech, Auburn, CA), and purity was determined by FACS analysis using anti-CD14-fluorescein isothiocyanate (FITC) (BioLegend, San Diego, CA). To generate immature DCs, $CD14^+$ monocytes were cultured in RPMI1640 supplemented with 10% human serum (Sigma) and 2 mM L-glutamine (Mediatech Inc) for six days in the presence of 25 ng/ml of human recombinant IL-4 (R&D Systems, Minneapolis, MN) and 25 ng/ml of human recombinant granulocyte-macrophage colony-stimulating factor (GM-CSF) (R&D Systems).

A total of 1.5×10^5 differentiated DCs was incubated with *H. pylori* lysates at a MOI of 5 for 48 hours in a 96-well plate. Similar treatment with 0.5 μg/ml lipopolysaccharide (LPS) (Sigma) served as a positive control. Pulsed DCs were gated out using anti-CD11c-phycoerythrin (PE) and analyzed for anti-CD40-allophycocyanin (APC), anti-CD86-Alexa Fluor 488, and anti-HLA-DR-peridinin chlorophyll protein complex (PerCP) (BioLegend) by flow cytometry. Acquisition was performed on a FACSCalibur (BD Biosciences), and data were analyzed using FlowJo software (Tree Star, Ashland, OR).

Chemical synthesis of αCGL, αCAG, αCPG and monoacylated αCPG

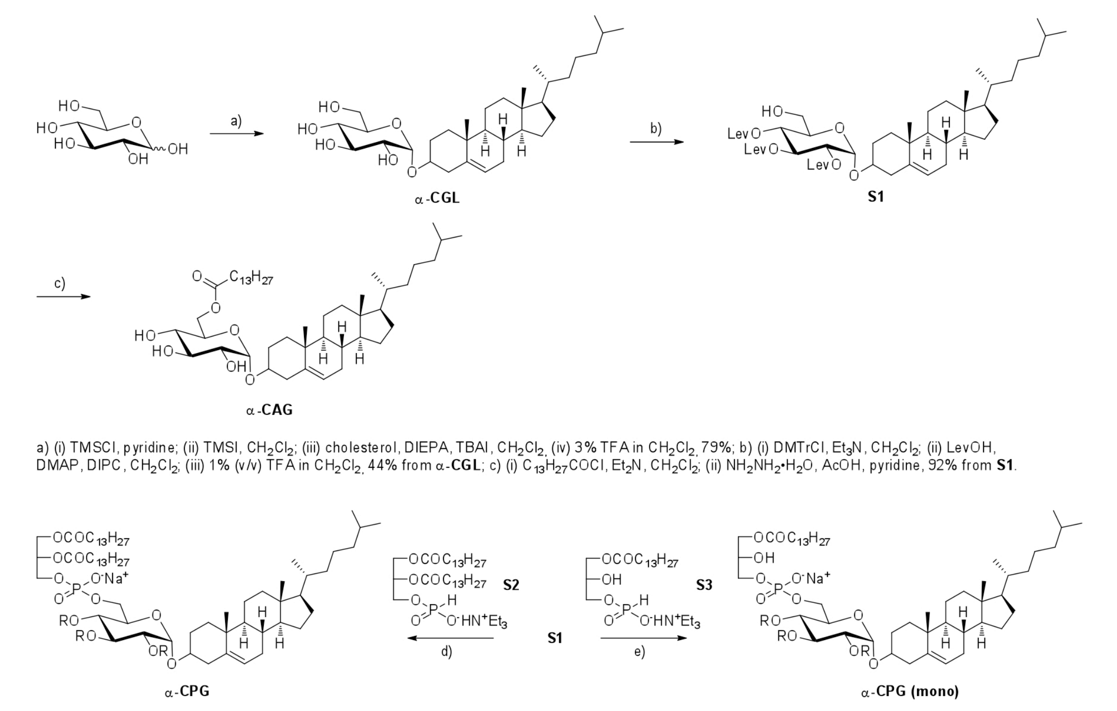

a) (i) TMSCl, pyridine; (ii) TMSI, CH_2Cl_2; (iii) cholesterol, DIEPA, TBAI, CH_2Cl_2, (iv) 3% TFA in CH_2Cl_2, 79%; b) (i) DMTrCl, Et_3N, CH_2Cl_2; (ii) LevOH, DMAP, DIPC, CH_2Cl_2; (iii) 1% (v/v) TFA in CH_2Cl_2, 44% from α-**CGL**; c) (i) $C_{13}H_{27}COCl$, Et_2N, CH_2Cl_2; (ii) NH_2NH_2•H_2O, AcOH, pyridine, 92% from **S1**.

d) (i) PivCl, pyridine; (ii) I_2, 5% H_2O (v/v) in pyridine; (iii) NH_2NH_2•H_2O, AcOH, pyridine; (iv) Dowex50WX8, 53% from **S1**.
e) (i) PivCl, pyridine; (ii) I_2, 5% H_2O (v/v) in pyridine; (iii) NH_2NH_2•H_2O, AcOH, pyridine; (iv) Dowex50WX8, 33% from **S1**.

NMR spectra were recorded on a JEOL ECX (400MHz) spectrometer in $CDCl_3$ or CD_3OD with tetramethylsilane (TMS) (δ 0.0) as an internal standard for ^1H NMR and $CDCl_3$ (δ 77.0) as an internal standard for ^{13}C NMR. ESI mass spectra were recorded on an Agilent ESI-TOF spectrometer. Chemicals and solvents were purchased from TCI America, Acros Organics, Alfa Aesar, Bachem and Sigma-Aldrich and used as supplied.

Cholesteryl α-D-glucopyranoside (αCGL). αCGL was synthesized using a method described previously [71]. Briefly, 2,3,4,6-tetrakis-*O*-(trimethylsilyl)-α-D-glucopyranosyl iodide was synthesized from D-glucose and reacted with cholesterol. Removal of trimethylsilyl group yielded **αCGL** as a colorless powder. ^1H NMR ($CDCl_3$-CD_3OD, 1:1, v/v) δ 5.35 (d, 1H, *J* = 3.7 Hz, C=C*H*), 4.98 (d, 1H, *J* = 3.7 Hz), 3.79 (m, 2H, H-6a, H-6b), 3.64–3.69 (m, 2H, H-3, H-5),

3.35–3.55 (m, 3H, H-2, H-4, H-3' cholesterol), 2.36 (m, 2H), 1.87–2.04 (m, 5H), 0.86–1.54 (m, 33H), 0.69 (s, 3H). ^{13}C NMR ($CDCl_3$-CD_3OD) δ 140.9, 122.3, 97.2, 76.4, 74.3, 72.4, 72.0, 70.6, 61.9, 57.1, 56.5, 50.5, 42.6, 40.3, 40.1, 39.8, 37.3, 37.0, 36.5, 36.1, 32.2, 28.5, 28.3, 27.0, 24.6, 24.1, 22.9, 22.7, 21.4, 19.5, 18.9, 12.0. ESI-MS calcd. for $[M+Na]^+$: 571.4; found 571.3.^{1}H NMR matched to the reported values [10].

Preparation of Cholesteryl 6-*O*-myristoyl-α-D-glucopyranoside (αCAG). 6-*O*-dimethoxytritylation and 2,3,4-tri-*O*-levulinylation to **α-CGL** were performed successively. Removal of the dimethoxytrityl group gave cholesteryl 2,4,6-tri-*O*-levulinyl-α-D-glucopyranoside (**S1**) as a colorless foam. (Data for **S1**) ^{1}H NMR ($CDCl_3$) δ 5.66 (t, 1H, *J* = 10 Hz, H-3), 5.33 (d, 1H, *J* = 5.5 Hz, C=C*H*), 5.16 (d, 1H, *J* = 3.7 Hz, H-1), 4.99 (t, 1H, *J* = 10 Hz, H-4), 4.80 (dd, 1H, *J* = 3.7 Hz, 10, H-2), 3.88 (m, 1H, H-5, H-6a), 3.74–3.59 (m, 2H, H-6b), 3.46–3.36 (m, 1H, H-3' cholesterol), 2.80–2.52 (m, 12H), 2.40–0.82 (m), 0.68 (s, 3H). ^{13}C NMR ($CDCl_3$) δ 206.5, 206.4, 206.2, 172.8, 171.8, 171.8, 140.3, 122.1, 94.2, 78.5, 71.1, 69.6, 69.5, 69.0, 60.8, 56.7, 56.1, 50.0, 42.3, 39.9, 39.7, 39.5, 37.8, 37.7, 37.6, 36.9, 36.6, 36.2, 35.7, 31.8, 29.8, 29.7, 29.7, 28.2, 28.0, 27.9, 27.8, 27.8, 27.7, 24.2, 23.8, 22.8, 22.5, 21.0, 19.3, 18.7, 11.8. ESI-MS calcd. for $[M + K]^+$: 881.5; found 881.5. 6-*O*-myristoylation of **S1** followed by removal of levulinyl group gave **αCAG** as a colorless foam. ^{1}H NMR ($CDCl_3$) δ 5.35 (m, 1H, C=C*H*), 5.02 (d, 1H, J = 4.3 Hz, H-1), 4.48 (dd, 1H, *J* = 1.8 Hz, 12 Hz, H-6a), 4.26 (dd, 1H, *J* = 1.8 Hz, 12 Hz, H-6b), 3.86 (m, 1H, H-5), 3.72 (t, 1H, J = 9.4 Hz, H-3), 3.54–3.44 (m, 2H, H-2, H-3' cholesterol), 3.33 (t, 1H, J = 9.4 Hz, H-4), 2.37–0.84 (m), 0.68 (s, 3H). ^{13}C NMR ($CDCl_3$) δ 174.7, 140.4, 122.3, 96.9, 78.5, 74.7, 72.2, 70.1, 63.2, 56.8, 56.2, 50.2, 42.3, 39.8, 39.6, 37.1,

36.7, 36.2, 35.9, 34.3, 32.0, 32.0, 29.8, 29.8, 29.7, 29.5, 29.4, 29.3, 28.3, 28.1, 25.1, 24.4, 24.0, 22.9, 22.8, 22.6, 21.1, 19.4, 18.8, 14.2, 11.9. ESI-MS calcd. for $[M+Na]^+$: 781.6; found 781.6. ^{1}H NMR matched to the reported value [10].

Preparation of Cholesteryl 6-*O*-(2',3'-bis-*O*-myristoyl-*rac*-glyceryl)phosphoryl-α-D-glucopyranoside sodium salt (αCPG) 1,2-Di-*O*-myristoyl-*rac*-glycerol was treated with PCl_3 and then 1 M aqueous triethylammonium bicarbonate to give **S2**, which was used without further purification. Condensation of **S2** and **S1** with pivaloyl chloride followed by oxidation with I_2 in pyridine–H_2O (95:5, v/v) and then removal of levulinyl groups gave αCPG as colorless foam. ^{1}H NMR (CD_3OD) δ 5.35 (br, 1H, C=C*H*), 5.24 (br, 1H, H-2'' glycerol), 4.97 (m, 1H, H-1), 4.44 (m, 1H, H-3'' glycerol), 4.29–4.16 (m, 2H, H-3'' glycerol, H-6a), 4.02–3.91 (m, 3H, H-6b, H-1'' glycerol), 3.70–3.65 (m, 2H, H-5, H-3), 3.57 (t, 1H, J = 9.2 Hz, H-4), 3.54–3.41 (m, 2H, H-2, H-3' cholesterol), 2.35–0.81 (m), 0.70 (s, 3H). HRMS (ESI) calcd. for $[M-H]^-$: 1121.7996; found 1121.7996.

Preparation of Cholesteryl 6-*O*-(3'-*O*-myristoyl-*rac*-glyceryl)phosphoryl-α-D-glucopyranoside sodium salt (αCPG (monoacyl)). 2-*O*-Levulinyl-3-*O*-myristoyl *rac*-glycerol was synthesized from DL-1,2-isopropylideneglycerol following a published method [72] and was treated with PCl_3 and then 1 M aqueous triethylammonium bicarbonate to give **S3**, which was used without further purification. Condensation of **S3** and **S1** with pivaloyl chloride followed by oxidation with I_2 in pyridine–H_2O (95:5, v/v) and then removal of levulinyl groups yielded αCPG (monoacyl) as a colorless powder. ^{1}H NMR (CD_3OD) δ 5.37 (br, 1H, C=C*H*),

4.97 (d, 1H, *J* = 3.1, H-1), 4.29 (m, 1H), 4.20–3.93 (m, 5H), 3.78–3.65 (m, 2H), 3.57–3.41 (m, 3H), 2.40–2.29 (m, 4H), 2.07–1.80 (m, 5H), 1.69–0.84 (m), 0.70 (s, 3H). HRMS (ESI) calcd. for $[M–H]^-$: 913.6164; found 913.6162.

Synthesis of βCGL: βCGL was synthesized using a method similar to that described previously [73].

In vitro **loading of lipid antigens and isoelectrofocusing analysis**

Mouse CD1d protein was expressed and purified as described [74]. Aliquots of 10 μg of mouse CD1d at a concentration of 20 μM were loaded overnight at room temperature in the presence of 3 times molar excess of each ligand (in DMSO, final concentration 3-6%) and 4mM CHAPS. Porcine brain sulfatide extract (Avanti Polar Lipid Inc) was used as positive control. The binding of charged lipids was assessed by isoelectric focusing on a PhastGel IEF 5-8 using a PhastSystem (GE Healthcare), followed by staining with Coomassie dye.

Total RNA extraction from mouse stomach and RT-PCR

Total RNA was isolated from half of a gastric tissue sample using TRIzol (Invtirogen, Carlsbad, CA). To remove genomic DNA, total RNA was digested with 10 U of RNase-free DNase I (New England Biolabs, Ipswich, MA) in the presence of 20 U of RNasin (Promega, Madison, WI) at 37°C for 3 hours. Three micrograms of DNase I treated-total RNA was reverse-transcribed using 100 U of Superscript II (Invitrogen) with 0.5 μg of oligo dT primer (Promega) at 42°C for 1 hour. PCR amplification of 0.5 μg of synthesized cDNA in a 10 μl reaction mixture was carried out in a GeneAmp 2700 thermal cycler (Applied Biosystems, Carlsbad, CA) using

0.5 U of Ex Taq HS (Takara Bio, Otsu, Japan) with 10 x PCR buffer and deoxynucleotide triphosphate at 0.2 mM and primers at 0.2 μM each. The primers, annealing temperature, and cycle conditions for Vα14Jα18, Foxp3, and β-actin are described in Table S2 [62-64]. Other primers for mice cytokines will be provided upon request.

Real-time PCR (Q-PCR) of human Vα24Jα18 mRNA

After measuring αCgT activity, 16 of 24 gastric biopsy specimens retrieved at Shinshu University Hospital were selected randomly. Total RNA was extracted from gastric biopsy specimens embedded in paraffin blocks with a minor modification as described [75]. Briefly, 10 slices of 5 μm-thick tissue sections were transferred to a 1.5 ml tube. DNase treated-total RNA was purified using a RecoverAll Total Nucleic Acid Isolation Kit (Ambion, Austin, TX) according to the manufacturer's instructions. For single-strand cDNA synthesis, 2 μg of total RNA, 0.5 μl of 0.5 μg/ μl Oligo(dT)$_{15}$ primer (Promega), and 0.5 μl of 0.5 μg/ μl Random primers (Promega) were incubated at 65ºC for 10 min to hybridize. Two micrograms of human stomach total RNA purchased from Clontech, Mountain View, CA, was used as a control. Total RNA was transcribed by adding 0.5 μl of 200 U/ μl SuperScript III Reverse Transcriptase (Invitrogen), 4 μl of 5x First-strand buffer, 1 μl of 2.5 mM dNTP mixture, 1 μl of 0.1 M dithiothreitol, and 0.5 μl of 40 U/ μl RNasin (Promega) at 50ºC for 60 min and then heated to 70ºC for 10 min. First-strand cDNA was used as template for Q-PCR.

Q-PCR was analyzed using a StepOnePlus Real Time PCR system (Applied Biosystems). The probe and primers for Vα24Jα18 were designed according to a published report [76]. The VIC-labeled probe was VIC- 5'-CCTCCTACATCTGTGTGGTGAGCGACA-

3'-MGB. The forward and reverse primers were 5'-CCTCCCAGCTCAGCGATTC-3', and 5'-TATAGCCTCCCCAGGGTTGA-3' respectively. A premixed FAM-labeled probe and primers for β_2-microglobulin (B2M) (Hs99999907_m1, Applied Biosystems) were used as an endogenous standard. The reaction was in 20 μl final volume containing 10 μl of TaqMan Gene Expression Master Mix (Applied Biosystems), 1 μl of cDNA, 250 nM of VIC or FAM-labeled TaqMan probe, and 900 nM of each primer in Fast 96-well reaction plates (Applied Biosystems). Plates were heated to 50ºC for 2 min then 95ºC for 10 min followed by 60 cycles at 95ºC for 15 sec, 55ºC for 1 min. Assays were performed in duplicate. Relative expression of Vα24Jα18 mRNA was determined with the comparative CT method using average CT values for Vα24Jα18 and B2M. Expression of Vα24Jα18 mRNA in commercially available total RNA from human stomach was assessed and defined as 1. Median values and the 25-75 percentile were indicated.

SUPPLEMENTAL REFERENCES

62. Dashtsoodol N, Watarai H, Sakata S, Taniguchi M (2008) Identification of CD4(-)CD8(-) double-negative natural killer T cell precursors in the thymus. PLoS One 3: e3688.

63. Kuwata K, Watanabe H, Jiang SY, Yamamoto T, Tomiyama-Miyaji C, et al. (2003) AIM inhibits apoptosis of T cells and NKT cells in Corynebacterium-induced granuloma formation in mice. Am J Pathol 162: 837-847.

64. Samon JB, Champhekar A, Minter LM, Telfer JC, Miele L, et al. (2008) Notch1 and TGFbeta1 cooperatively regulate Foxp3 expression and the maintenance of peripheral regulatory T cells. Blood 112: 1813-1821.

65. Segal ED (1995) Electroporation of Helicobacter pylori. Methods Mol Biol 47: 179-184.

66. Pei J, Grishin NV (2007) PROMALS: towards accurate multiple sequence alignments of distantly related proteins. Bioinformatics 23: 802-808.

67. Arnold K, Bordoli L, Kopp J, Schwede T (2006) The SWISS-MODEL workspace: a web-based environment for protein structure homology modelling. Bioinformatics 22: 195-201.

68. Kleywegt GJ, Jones TA (1998) Databases in protein crystallography. Acta Crystallogr D Biol Crystallogr 54: 1119-1131.

69. Emsley P, Cowtan K (2004) Coot: model-building tools for molecular graphics. Acta Crystallogr D Biol Crystallogr 60: 2126-2132.

70. Brunger AT, Adams PD, Clore GM, DeLano WL, Gros P, et al. (1998) Crystallography & NMR system: A new software suite for macromolecular structure determination. Acta Crystallogr D Biol Crystallogr 54: 905-921.

71. Kulkarni SS, Gervay-Hague J (2008) Two-step synthesis of the immunogenic bacterial glycolipid BbGL1. Org Lett 10: 4739-4742.

72. Greimel PI, Y. (2008) First synthesis of natural phosphatidyl-β- d-glucoside. Tetrahedron Letters 49: 3562–3566.

73. Ben-Menachem G, Kubler-Kielb J, Coxon B, Yergey A, Schneerson R (2003) A newly discovered cholesteryl galactoside from Borrelia burgdorferi. Proc Natl Acad Sci U S A 100: 7913-7918.

74. Wang J, Li Y, Kinjo Y, Mac TT, Gibson D, et al. (2010) Lipid binding orientation within CD1d affects recognition of Borrelia burgorferi antigens by NKT cells. Proc Natl Acad Sci U S A 107: 1535-1540.

75. Ito Y, Watanabe M, Nishizawa T, Omachi T, Kobayashi T, et al. (2007) The utility of formalin-fixed and paraffin-embedded tissue blocks for quantitative analysis of N-acetylgalactosamine 4-sulfate 6-O-sulfotransferase mRNA expressed by colorectal cancer cells. Acta Histochem Cytochem 40: 53-59.

76. Metelitsa LS, Wu HW, Wang H, Yang Y, Warsi Z, et al. (2004) Natural killer T cells infiltrate neuroblastomas expressing the chemokine CCL2. J Exp Med 199: 1213-1221.
